# A novel phytocannabinoid isolated from *Cannabis sativa* L. with an *in vivo* cannabimimetic activity higher than Δ^9^-tetrahydrocannabinol: Δ^9^-Tetrahydrocannabiphorol

**DOI:** 10.1038/s41598-019-56785-1

**Published:** 2019-12-30

**Authors:** Cinzia Citti, Pasquale Linciano, Fabiana Russo, Livio Luongo, Monica Iannotta, Sabatino Maione, Aldo Laganà, Anna Laura Capriotti, Flavio Forni, Maria Angela Vandelli, Giuseppe Gigli, Giuseppe Cannazza

**Affiliations:** 1 Mediteknology spin-off company of the National Council of Research (CNR), Via Arnesano, 73100 Lecce, Italy; 2 Institute of Nanotechnology of the National Council of Research (CNR NANOTEC), Via Monteroni, 73100 Lecce, Italy; 3 Department of Life Sciences, University of Modena and Reggio Emilia, Via Campi 103, 41125 Modena, Italy; 4 Department of Experimental Medicine, Division of Pharmacology, Università della Campania “L. Vanvitelli”, Via Santa Maria di Costantinopoli 16, 80138 Naples, Italy; 5 Department of Chemistry, Sapienza University of Rome, Piazzale Aldo Moro 5, 00185 Rome, Italy

**Keywords:** Biological techniques, Drug discovery, Plant sciences, Chemistry

## Abstract

(-)-*Trans*-Δ^9^-tetrahydrocannabinol (Δ^9^-THC) is the main compound responsible for the intoxicant activity of *Cannabis sativa* L. The length of the side alkyl chain influences the biological activity of this cannabinoid. In particular, synthetic analogues of Δ^9^-THC with a longer side chain have shown cannabimimetic properties far higher than Δ^9^-THC itself. In the attempt to define the phytocannabinoids profile that characterizes a medicinal cannabis variety, a new phytocannabinoid with the same structure of Δ^9^-THC but with a seven-term alkyl side chain was identified. The natural compound was isolated and fully characterized and its stereochemical configuration was assigned by match with the same compound obtained by a stereoselective synthesis. This new phytocannabinoid has been called (-)-*trans*-Δ^9^-tetrahydrocannabiphorol (Δ^9^-THCP). Along with Δ^9^-THCP, the corresponding cannabidiol (CBD) homolog with seven-term side alkyl chain (CBDP) was also isolated and unambiguously identified by match with its synthetic counterpart. The binding activity of Δ^9^-THCP against human CB_1_ receptor *in vitro* (K_i_ = 1.2 nM) resulted similar to that of CP55940 (K_i_ = 0.9 nM), a potent full CB_1_ agonist. In the cannabinoid tetrad pharmacological test, Δ^9^-THCP induced hypomotility, analgesia, catalepsy and decreased rectal temperature indicating a THC-like cannabimimetic activity. The presence of this new phytocannabinoid could account for the pharmacological properties of some cannabis varieties difficult to explain by the presence of the sole Δ^9^-THC.

## Introduction

*Cannabis sativa* has always been a controversial plant as it can be considered as a lifesaver for several pathologies including glaucoma^1^ and epilepsy^2^, an invaluable source of nutrients^3^, an environmentally friendly raw material for manufacturing^4^ and textiles^5^, but it is also the most widely spread illicit drug in the world, especially among young adults^6^.

Its peculiarity is its ability to produce a class of organic molecules called phytocannabinoids, which derive from an enzymatic reaction between a resorcinol and an isoprenoid group. The modularity of these two parts is the key for the extreme variability of the resulting product that has led to almost 150 different known phytocannabinoids^7^. The precursors for the most commonly naturally occurring phytocannabinoids are olivetolic acid and geranyl pyrophosphate, which take part to a condensation reaction leading to the formation of cannabigerolic acid (CBGA). CBGA can be then converted into either tetrahydrocannabinolic acid (THCA) or cannabidiolic acid (CBDA) or cannabichromenic acid (CBCA) by the action of a specific cyclase enzyme^7^. All phytocannabinoids are biosynthesized in the carboxylated form, which can be converted into the corresponding decarboxylated (or neutral) form by heat^8^. The best known neutral cannabinoids are undoubtedly Δ^9^-tetrahydrocannabinol (Δ^9^-THC) and cannabidiol (CBD), the former being responsible for the intoxicant properties of the cannabis plant, and the latter being active as antioxidant, anti-inflammatory, anti-convulsant, but also as antagonist of THC negative effects^9^.

All these cannabinoids are characterized by the presence of an alkyl side chain on the resorcinyl moiety made of five carbon atoms. However, other phytocannabinoids with a different number of carbon atoms on the side chain are known and they have been called varinoids (with three carbon atoms), such as cannabidivarin (CBDV) and Δ^9^-tetrahydrocannabivarin (Δ^9^-THCV), and orcinoids (with one carbon atom), such as cannabidiorcol (CBD-C_1_) and tetrahydrocannabiorcol (THC-C_1_)^7^. Both series are biosynthesized in the plant as the specific ketide synthases have been identified^10^.

Our research group has recently reported the presence of a butyl phytocannabinoid series with a four-term alkyl chain, in particular cannabidibutol (CBDB) and Δ^9^-tetrahydrocannabutol (Δ^9^-THCB), in CBD samples derived from hemp and in a medicinal cannabis variety^11,12^. Since no evidence has been provided for the presence of plant enzymes responsible for the biosynthesis of these butyl phytocannabinoids, it has been suggested that they might derive from microbial ω-oxidation and decarboxylation of their corresponding five-term homologs^13^.

The length of the alkyl side chain has indeed proved to be the key parameter, the pharmacophore, for the biological activity exerted by Δ^9^-THC on the human cannabinoid receptor CB_1_ as evidenced by structure-activity relationship (SAR) studies collected by Bow and Rimondi^14^. In particular, a minimum of three carbons is necessary to bind the receptor, then the highest activity has been registered with an eight-carbon side chain to finally decrease with a higher number of carbon atoms^14^. Δ^8^-THC homologs with more than five carbon atoms on the side chain have been synthetically produced and tested in order to have molecules several times more potent than Δ^9^-THC^15,16^.

To the best of our knowledge, a phytocannabinoid with a linear alkyl side chain containing more than five carbon atoms has never been reported as naturally occurring. However, our research group disclosed for the first time the presence of seven-term homologs of CBD and Δ^9^-THC in a medicinal cannabis variety, the Italian FM2, provided by the Military Chemical Pharmaceutical Institute in Florence. The two new phytocannabinoids were isolated and fully characterized and their absolute configuration was confirmed by a stereoselective synthesis. According to the International Non-proprietary Name (INN), we suggested for these CBD and THC analogues the name “cannabidiphorol” (CBDP) and “tetrahydrocannabiphorol” (THCP), respectively. The suffix “-phorol” comes from “sphaerophorol”, common name for 5-heptyl-benzen-1,3-diol, which constitutes the resorcinyl moiety of these two new phytocannabinoids.

A number of clinical trials^17–19^ and a growing body of literature provide real evidence of the pharmacological potential of cannabis and cannabinoids on a wide range of disorders from sleep to anxiety, multiple sclerosis, autism and neuropathic pain^20–23^. In particular, being the most potent psychotropic cannabinoid, Δ^9^-THC is the main focus of such studies. In light of the above and of the results of the SAR studies^14–16^, we expected that THCP is endowed of an even higher binding affinity for CB_1_ receptor and a greater cannabimimetic activity than THC itself. In order to investigate these pharmacological aspects of THCP, its binding affinity for CB_1_ receptor was tested by a radioligand *in vitro* assay and its cannabimimetic activity was assessed by the tetrad behavioral tests in mice.

## Results

### Identification of cannabidiphorol (CBDP) and Δ^9^-tetrahydrocannabiphorol (Δ^9^-THCP) by liquid chromatography coupled to high-resolution mass spectrometry (LC-HRMS)

**Figure 1:**
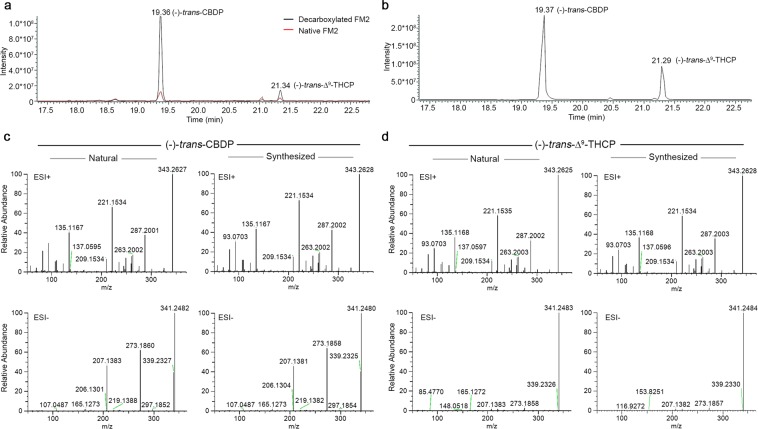
UHPLC-HRMS identification of (-)-*trans*-CBDP and (-)-*trans*-Δ^9^-THCP. Extracted ion chromatograms (EIC) of CBDP and Δ^9^-THCP from a standard mixture at 25 and 10 ng/mL respectively (**a**) and from the native (red plot) and decarboxylated (black plot) FM2 (**b**). (**c,d)** Comparison of the high-resolution fragmentation spectra of synthetic and natural CBDP and Δ^9^-THCP in both positive (ESI+) and negative (ESI−) mode.

The FM2 ethanolic extract was analyzed by an analytical method recently developed for the cannabinoid profiling of this medicinal cannabis variety^12,24^. As the native extract contains mainly the carboxylated forms of phytocannabinoids as a consequence of a cold extraction^25^, part of the plant material was heated to achieve decarboxylation where the predominant forms are neutral phytocannabinoids. The advanced analytical platform of ultra-high performance liquid chromatography coupled to high resolution Orbitrap mass spectrometry was employed to analyze the FM2 extracts and study the fragmentation spectra of the analytes under investigation. The precursor ions of the neutral derivatives cannabidiphorol (CBDP) and Δ^9^-tetrahydrocannabiphorol (Δ^9^-THCP), 341.2486 for the [M-H]^−^ and 343.2632 for the [M + H]^+^, showed an elution time of 19.4 min for CBDP and 21.3 min for Δ^9^-THCP (Fig. 1a). Their identification was confirmed by the injection of a mixture (5 ng/mL) of the two chemically synthesized CBDP and Δ^9^-THCP (Fig. 1b) as it will be described later. As for their carboxylated counterpart, the precursor ions of the neutral forms CBDP and Δ^9^-THCP break in the same way in ESI+ mode, but they show a different fragmentation pattern in ESI− mode. Whilst Δ^9^-THCP shows only the precursor ion [M-H]^−^ (Fig. 1d), CBDP molecule generates the fragments at *m/z* 273.1858 corresponding to a retro Diels-Alder reaction, and 207.1381 corresponding to the resorcinyl moiety after the break of the bond with the terpenoid group (Fig. 1c). It is noteworthy that for both molecules, CBDP and Δ^9^-THCP, each fragment in both ionization modes differ exactly by an ethylene unit (CH_2_)_2_ from the corresponding five-termed homologs CBD and THC. Moreover, the longer elution time corroborates the hypothesis of the seven-termed phytocannabinoids considering the higher lipophilicity of the latter.

### Isolation and characterization of natural CBDP and Δ^9^-THCP

In order to selectively obtain a cannabinoid-rich fraction of FM2, *n*-hexane was used to extract the raw material instead of ethanol, which carries other contaminants such as flavonoids and chlorophylls along with cannabinoids^26^. An additional dewaxing step at −20 °C for 48 h and removal of the precipitated wax was necessary to obtain a pure cannabinoids extract. Semi-preparative liquid chromatography with a C_18_ stationary phase allowed for the separation of 80 fractions, which were analyzed by LC-HRMS with the previously described method. In this way, the fractions containing predominantly cannabidiphorolic acid (CBDPA) and tetrahydrocannabipgorolic acid (THCPA) were separately subject to heating at 120 °C for 2 h in order to obtain their corresponding neutral counterparts CBDP and Δ^9^-THCP as clear oils with a >95% purity. The material obtained was sufficient for a full characterization by ^1^H and ^13^C NMR, circular dichroism (CD) and UV absorption.

### Stereoselective synthesis of CBDP and Δ^9^-THCP

**Figure 2:**
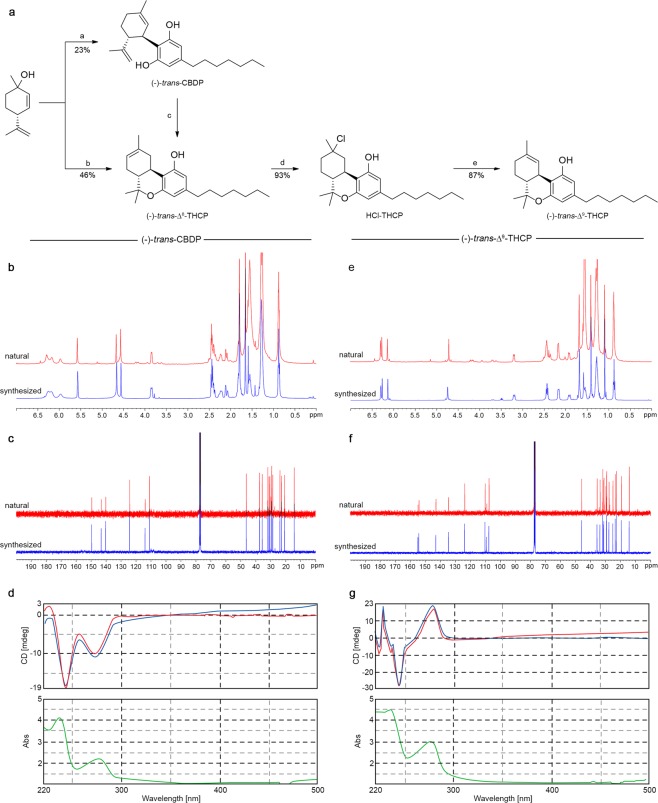
Synthesis and spectroscopic characterization of (-)-*trans*-CBDP and (-)-*trans*-Δ^9^-THCP. (**a**) Reagents and conditions: (*a*) 5-heptylbenzene-1,3-diol (1.1 eq.), *p*TSA (0.1 eq.), CH_2_Cl_2_, r.t., 90 min.; (*b*) 5-heptylbenzene-1,3-diol (1.1 eq.), *p*TSA (0.1 eq.), DCM, r.t., 48 h; (*c*) *p*TSA (0.1 eq.), DCM, r.t., 48 h; (*d*) ZnCl_2_ (0.5 eq.), 4 N HCl in dioxane (1 mL per 100 mg of Δ^8^-THCP), dry DCM, argon, 0 °C to r.t., 2 h. (*e*) 1.75 M potassium *t-*amylate in toluene (2.5 eq.), dry toluene, argon, −15 °C, 1 h. (**b–g)** Superimposition of ^1^H, ^13^C NMR and CD spectra for natural (red line) and synthesized (blue line) (-)-*trans*-CBDP (**b–d**) and (-)-*trans*-Δ^9^-THCP (**e–g**).

(-)-*trans*-Cannabidiphorol ((-)-*trans*-CBDP) and (-)-*trans*-Δ^9^-tetrahydrocannabiphorol ((-)-*trans*-Δ^9^-THCP) were stereoselectively synthesized as previously reported for the synthesis of (-)-*trans*-CBDB and (-)-*trans*-Δ^9^-THCB homologs^11,12,24^. Accordingly, (-)-*trans*-CBDP was prepared by condensation of 5-heptylbenzene-1,3-diol with (1 *S*,4 *R*)-1-methyl-4-(prop-1-en-2-yl)cycloex-2-enol, using *p*TSA as catalyst, for 90 min. Longer reaction time did not improve the yield of (-)-*trans*-CBDP because cyclization of (-)-*trans*-CBDP to (-)-*trans*-Δ^9^-THCP and then to (-)-*trans*-Δ^8^-THCP occurred. 5-heptylbenzene-1,3-diol was synthesized first as reported in the Supporting Information (Supplementary Fig. SI-1). The conversion of (-)-*trans*-CBDP to (-)-*trans*-Δ^9^-THCP using diverse Lewis’ acids, as already reported in the literature for the synthesis of the homolog Δ^9^-THC^27–29^, led to a complex mixture of isomers which resulted in an arduous and low-yield isolation of (-)-*trans*-Δ^9^-THCP by standard chromatographic techniques. Therefore, for the synthesis of (-)-*trans*-Δ^9^-THCP, its regioisomer (-)-*trans*-Δ^8^-THCP was synthesized first by condensation of 5-heptylbenzene-1,3-diol with (1 *S*,4 *R*)-1-methyl-4-(prop-1-en-2-yl)cycloex-2-enol, as described above, but the reaction was left stirring for 48 hours. Alternatively, (-)-*trans*-CBDP could be also quantitatively converted to (-)-*trans*-Δ^8^-THCP in the same conditions. Hydrochlorination of the Δ^8^ double bond of (-)-*trans*-Δ^8^-THCP, using ZnCl_2_ as catalyst, allowed to obtain (-)-*trans*-HCl-THCP, which was successively converted to (-)-*trans*-Δ^9^-THCP in 87% yield by selective elimination on position 2 of the terpene moiety using potassium *t*-amylate as base (Fig. 2a).

The chemical identification of synthetic (-)-*trans*-CBDP and (-)-*trans*-Δ^9^-THCP, and their unambiguous ^1^H and ^13^C assignments were achieved by NMR spectroscopy (Supplementary Table SI-1,2 and Supplementary Fig. SI-2,3). Since (-)-*trans*-CBDP and (-)-*trans*-Δ^9^-THCP differ from the respective homologs (CBD, CBDB, CBDV, Δ^9^-THC, Δ^9^-THCB and Δ^9^-THCV) solely for the length of the alkyl chain on the resorcinyl moiety, no significant differences in the proton chemical shifts of the terpene and aromatic moieties were observed for CBD and Δ^9^-THC homologs. The perfect match in the chemical shift of the terpene and aromatic moieties between the synthesized (-)-*trans*-CBDP and (-)-*trans*-Δ^9^-THCP and the respective homologues^11,24,30^, combined with the mass spectra and fragmentation pattern, allowed us to unambiguously confirm the chemical structures of the two new synthetic cannabinoids. The *trans* (1*R*,6*R*) configuration at the terpene moiety was confirmed by optical rotatory power. The new cannabinoids (-)-*trans*-CBDP and (-)-*trans*-Δ^9^-THCP showed an [α]_D_^20^ of −145° and −166°, respectively, in chloroform. The [α]_D_^20^ values were in line with those of the homologs^11,31^, suggesting a (1*R*,6*R*) configuration for both CBDP and Δ^9^-THCP. A perfect superimposition between the ^1^H (Fig. 2b,e) and ^13^C NMR spectra (Fig. 2c,f) and the circular dichroism absorption (Fig. 2d,g) of both synthetic and extracted (-)-*trans*-CBDP and (-)-*trans*-Δ^9^-THCP was observed, confirming the identity of the two new cannabinoids identified in the FM2 cannabis variety.

### Binding affinity at human CB_1_ and CB_2_ receptors

**Figure 3:**
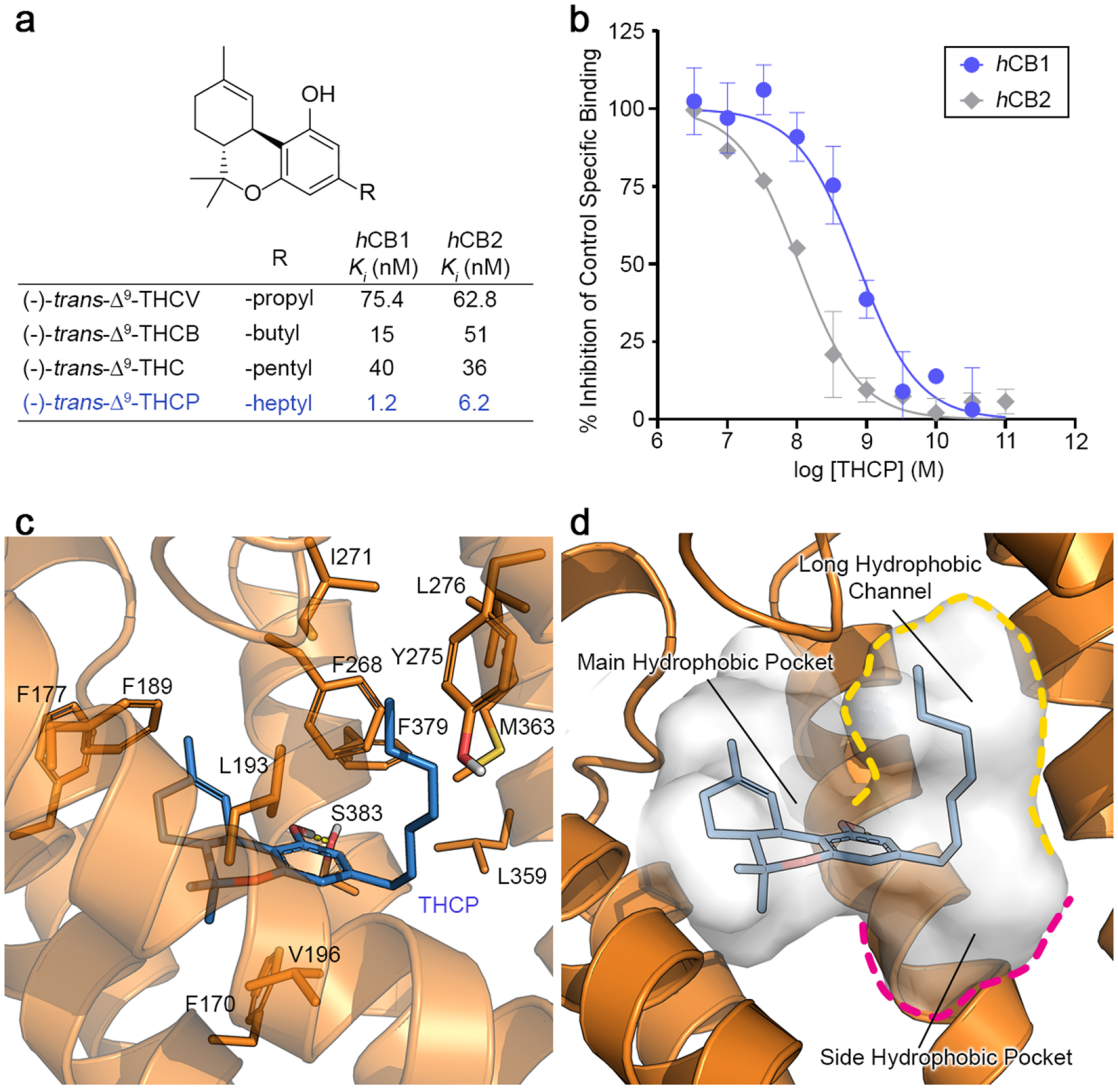
*In vitro* activity and docking calculation of Δ^9^-THCP. (**a**) Binding affinity (*K*_*i*_) of the four homologues of Δ^9^-THC against human CB_1_ and CB_2_ receptors. (**b**) Dose-response studies of Δ^9^-THCP against *h*CB_1_ (in blue) and *h*CB_2_ (in grey). All experiments were performed in duplicate and error bars denote s.e.m. of measurements. (**c**) Docking pose of (-)-*trans*-Δ^9^-THCP (blue sticks), in complex with *h*CB_1_ receptor (PDB ID: 5XRA, orange cartoon). Key amino acidic residues are reported in orange sticks. H-bonds are reported in yellow dotted lines. Heteroatoms are color-coded: oxygen in red, nitrogen in blue and sulphur in yellow. (**d**) Binding pocket of *h*CB_1_ receptor, highlighting the positioning of the heptyl chain within the long hydrophobic channel of the receptor (yellow dashed line). The side hydrophobic pocket is bordered in magenta. Panels **c** and **d** were built using Maestro 10.3 of the Schrödinger Suite.

The binding affinity of (-)-*trans*-Δ^9^-THCP against purified human CB_1_ and CB_2_ receptors was determined in a radioligand binding assay, using [^3^H]CP55940 or [^3^H]WIN 55212-2 as reference compounds, and dose-response curves were determined (Fig. 3a,b). (-)-*trans*-Δ^9^-THCP binds with high affinity to both human CB_1_ and CB_2_ receptors with a *K*_*i*_ of 1.2 and 6.2 nM, respectively. (-)-*trans*-Δ^9^-THCP resulted 33-times more active than (-)-*trans*-Δ^9^-THC (*K*_*i*_ = 40 nM), 63-times more active than (-)-*trans*-Δ^9^-THCV (*K*_*i*_ = 75.4 nM) and 13-times more active than the newly discovered (-)-*trans*-Δ^9^-THCB (*K*_*i*_ = 15 nM) against CB_1_ receptor^12,14^. Moreover, the new identified (-)-*trans*-Δ^9^-THCP resulted about 5- to 10-times more active against CB_2_ receptor (*K*_*i*_ = 6.2 nM), in contrast with (-)-*trans*-Δ^9^-THC, (-)-*trans*-Δ^9^-THCB and (-)-*trans*-Δ^9^-THCV, which instead showed a comparable binding affinity with a *K*_*i*_ ranging from 36 to 63 nM (Fig. 3a)^12,14^.

The highest activity of (-)-*trans*-Δ^9^-THCP, compared to the shorter homologues, was investigated by docking calculation. The X-ray structure of the active conformation of *h*CB_1_ receptor in complex with the agonist AM11542 (PDB ID: 5XRA) was used as reference for docking since marked structural changes in the orthosteric ligand-binding site are observed in comparison with the conformation of the receptor bound to an antagonist^32,33^. AM11542 is a synthetic Δ^8^ cannabinoid with high affinity against *h*CB_1_ receptor (*K*_*i*_ = 0.11 nM) possessing a 7′-bromo-1′,1′-dimethyl-heptyl aliphatic chain at C3 of the resorcinyl moiety. As expected, due to the close chemical similarity, the predicted binding mode of (-)-*trans*-Δ^9^-THCP (Fig. 3c) reflected that of AM11542 in the CB_1_ crystal structure (Fig. SI-6a,b)^18^. (-)-*trans*-Δ^9^-THCP bound in the active conformation of CB_1_ in an L-shaped pose. The tetrahydro-6*H*-benzo[c]chromene ring system is located within the main hydrophobic pocket delimited by Phe174, Phe177, Phe189, Lys193, Pro269, Phe170, and Phe268. In particular, the aromatic ring of the resorcinyl moiety is involved in two edge-to-face π-π interactions with Phe170 and Phe268, whereas the phenolic hydroxyl group at C1 is engaged in a H-bond with Ser383 (Fig. 3c). Interestingly, the heptyl chain at C3 extended into a long hydrophobic tunnel formed by Leu193, Val196, Tyr275, Iso271, Leu276, Trp279, Leu359, Phe379, and Met363 (Fig. 3c,d). Because the predicted pose of the tricyclic tetrahydrocannabinol ring system is conserved among the four THC homologs (Supplementary Fig. SI-7a–c), the length of the alkyl chain at C3 of the resorcinyl moiety could account for the different binding affinity observed among the four cannabinoids. (-)-*trans*-Δ^9^-THCP (Fig. 3c) and (-)-*trans*-Δ^9^-THC (Supplementary Fig. SI-7a) share the same positioning of the alkyl ‘tail’ within the hydrophobic channel^12,33,34^. However, the long heptyl chain of Δ^9^-THCP is able to extend into the tunnel along its entire length, maximizing the hydrophobic interactions with the residues of the side channel. In contrast, the tunnel is only partially occupied by the shorter pentyl chain of (-)-*trans*-Δ^9^-THC, accounting for the higher affinity of Δ^9^-THCP (*K*_*i*_ = 1.2 nM) compared to Δ^9^-THC (*K*_*i*_ = 40 nM). A different positioning of the ‘tail’ was instead predicted for the shorter alkyl chain homologues, Δ^9^-THCV and Δ^9^-THCB. The propyl and butyl chain of Δ^9^-THCV and Δ^9^-THCB, respectively, are too short to effectively extend within the hydrophobic channel. As stated in our previous work^12^, these shorter chains accommodate within a small hydrophobic pocket delimitated by Phe200, Leu359, and Met363 (Supplementary Fig. SI–7b,c). This side pocket is located at the insertion between the main hydrophobic pocket and the long channel (Fig. 3d) and seems to accommodate small hydrophobic substituents (i.e. gem-dimethyl or cycloalkyl) introduced at C1′ position of the side chain of several synthetic cannabinoids, rationalizing the notable enhancement in potency and affinity for these derivatives^35–39^.

### *In vivo* determination of the cannabinoid profile of Δ^9^-THCP

**Figure 4:**
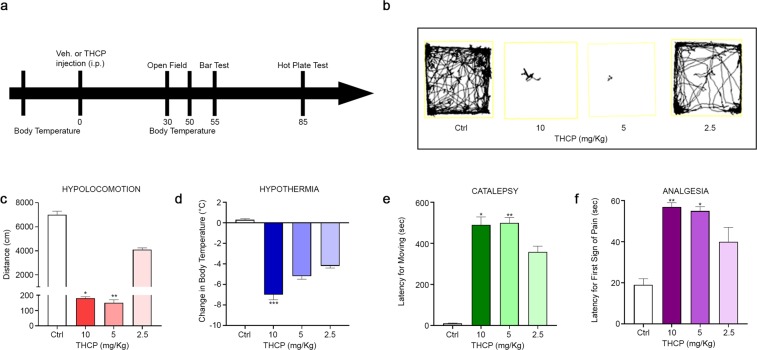
Dose-dependent effects of Δ^9^-THCP administration (2.5, 5, or 10 mg/kg, i.p.) on the tetrad phenotypes in mice in comparison to vehicle. (**a**) Time schedule of the tetrad tests in minutes from Δ^9^-THCP or vehicle administration. (**b,c**) Locomotion decrease induced by Δ^9^-THCP administration in the open field test. (**d**) Decrease of body temperature after Δ^9^-THCP administration; the values are expressed as the difference between the basal temperature (i.e., taken before Δ^9^-THCP or vehicle administration) and the temperature measured after Δ^9^-THCP or vehicle administration. (**e**) Increase in the latency for moving from the catalepsy bar after Δ^9^-THCP administration. (**f**) Increase in the latency after the first sign of pain shown by the mouse in the hot plate test following Δ^9^-THCP administration. Data are represented as mean ± SEM of 5 mice per group. * indicate significant differences compared to 0 (vehicle injection), respectively. *p < 0.05, **p < 0.01, ***p < 0.001 versus Δ^9^-THCP 0 mg/kg (vehicle). The Kruskall-Wallis test followed by Dunn’s post hoc tests were performed for statistical analysis.

The cannabinoid activity of Δ^9^-THCP was evaluated by the tetrad of behavioural tests on mice. The tetrad includes the assessment of spontaneous activity, immobility index (catalepsy), analgesia and changes in rectal temperature. Decrease of locomotor activity, catalepsy, analgesia and hypothermia are well-known signs of physiological manifestations of cannabinoid activity^40^. After intraperitoneal (i.p.) administration, Δ^9^-THCP at 2.5 mg/kg markedly reduced the spontaneous activity of mice in the open field, while at 5 and 10 mg/kg it induced catalepsy on the ring with the immobility as compared to the vehicle treated mice (Fig. 4b,c) (0 mg/kg: 6888 cm ± 474.8, 10 mg/kg: 166.8 cm ± 20.50, 5 mg/kg: 127.5 cm ± 31.32, 2.5 mg/kg: 4072 cm ± 350.8, p = 0.0009). Moreover, Δ^9^-THCP administration induced a significant increase, at 10 and 5 mg/kg, in the latency for moving from the catalepsy bar (Fig. 4e) (0 mg/kg: 15.20 sec ± 4.33, 10 mg/kg: 484.5 sec ± 51.58, 5 mg/kg: 493.4 sec ± 35.68, 2.5 mg/kg: 346.1 sec ± 35.24, p = 0.0051). In the hot plate test (Fig. 4f), Δ^9^-THCP (10 and 5 mg/kg) induced antinociceptive effect, whereas at 2.5 mg/kg there was a trend in the induction of antinociception, which resulted not statistically significant as compared to the vehicle treated mice (0 mg/kg: 19.20 sec ± 2.65, 10 mg/kg: 57.0 sec ± 2.0, 5 mg/kg: 54.38 sec ± 2.86, 2.5 mg/kg: 40.22 sec ± 5.8, p = 0.0044). Δ^9^-THCP administration induced a dose dependent significant decrease, only at 10 mg/kg, in body temperature as compared to vehicle (0 mg/kg: 0.40 °C ± 0.25, 10 mg/kg: −7.10 °C ± 0.43, 5 mg/kg: −5.28 °C ± 0.36, 2.5 mg/kg: −4,12 °C ± 0.38, p = 0.0009) (Fig. 4d).

### Semi-quantification of CBDP and Δ^9^-THCP in the FM2 extract

A semi-quantification method based on LC-HRMS allowed to provide an approximate amount of the two new phytocannabinoids in the FM2 ethanol extract. Their pentyl homologues, CBD and Δ^9^-THC, showed a concentration of 56 and 39 mg/g respectively, in accordance with the values provided by the Military Chemical Pharmaceutical Institute (59 mg/g and 42 mg/g for CBD and Δ^9^-THC respectively), obtained by the official GC-FID quantitative method. The same semi-quantitative method provided an amount of about 243 and 29 µg/g for CBDP and Δ^9^-THCP respectively.

## Discussion

Up to now, almost 150 phytocannabinoids have been detected in cannabis plant^7,41,42^, though most of them have neither been isolated nor characterized. The well-known CBD and Δ^9^-THC have been extensively characterized and proved to possess interesting pharmacological profiles^43–47^, thus the attention towards the biological activity of their known homologs like CBDV and Δ^9^-THCV has recently grown as evidenced by the increasing number of publications per year appearing on Scopus. Other homologs like those belonging to the orcinoid series are scarcely investigated likely due to their very low amount in the plant that makes their isolation very challenging. In recent years, the agricultural genetics research has made great progresses on the selection of rare strains that produce high amounts of CBDV, CBG and Δ^9^-THCV^48–50^, thus it would not be surprising to see in the near future cannabis varieties rich in other minor phytocannabinoids. This genetic selection would enable the production of extracts rich in a specific phytocannabinoid with a characteristic pharmacological profile. For this reason, it is important to carry out a comprehensive chemical profiling of a medicinal cannabis variety and a thorough investigation of the pharmacological activity of minor and less known phytocannabinoids.

As the pharmacological activity of Δ^9^-THC is particularly ascribed to its affinity for CB_1_ receptor, the literature suggests that the latter can be increased by elongating the alkyl side chain, which represents the main cannabinoid pharmacophoric driving force^14^. Therefore, taking THC as the lead compound, a series of cannabinoids have been chemically synthesized and their biological potency resulted several times higher than Δ^9^-THC itself^15^. To the best of our knowledge, naturally occurring cannabinoids with a linear alkyl side chain longer than five terms have never been detected or even putatively identified in cannabis plant. However, the cutting-edge technological platform of the Orbitrap mass spectrometry and the use of advanced analytical techniques like metabolomics can enable the discovery and identification of new compounds with a high degree of confidence even when present in traces in complex matrices^42,51^. In the present work, we report for the first time the isolation and full characterization of two new CBD and Δ^9^-THC heptyl homologs, which we named cannabidiphorol (CBDP) and Δ^9^-tetrahydrocannabiphorol (Δ^9^-THCP), respectively. These common names were derived from the traditional naming of phytocannabinoids based on the resorcinyl residue, in this case corresponding to sphaerophorol.

The biological results obtained in the *in vitro* binding assay indicated an affinity for CB_1_ receptor more than thirty-fold higher compared to the one reported for Δ^9^-THC in the literature^14^. Also, this encouraging data was supported by *in vivo* evaluation of the cannabimimetic activity by the tetrad test, where Δ^9^-THCP decreased locomotor activity and rectal temperature, induced catalepsy and produced analgesia miming the properties of full CB_1_ receptor agonists (Fig. 4). In particular, Δ^9^-THCP proved to be as active as Δ^9^-THC but at lower doses. In fact, the minimum THC dose used in this kind of test is 10 mg/kg, whereas Δ^9^-THCP resulted active at 5 mg/kg in three of the four tetrad tests. These results, accompanied by the docking data, are in line with the extensive structure-activity relationship (SAR) studies performed through the years on synthetic cannabinoids, revealing the importance of the length of the alkyl chain in position 3 on the resorcinyl moiety in modulating the ligand affinity at CB_1_ receptor.

Although the amount of the heptyl homologues of CBD and Δ^9^-THC in the FM2 variety could appear trifling, both *in vitro* and *in vivo* preliminary studies reported herein on Δ^9^-THCP showed a cannabimimetic activity several times higher than its pentyl homolog Δ^9^-THC. Moreover, it is reasonable to suppose that other cannabis varieties may contain even higher percentages of Δ^9^-THCP. It is also important to point out that there exists an astonishing variability of subject response to a cannabis-based therapy even with an equal Δ^9^-THC dose^52–54^. It is therefore possible that the psychotropic effects are due to other extremely active phytocannabinoids such as Δ^9^-THCP. However, up to now nobody has ever searched for this potent phytocannabinoid in medicinal cannabis varieties. In our opinion, this compound should be included in the list of the main phytocannabinoids to be determined for a correct evaluation of the pharmacological effect of the cannabis extracts administered to patients. In fact, we believe that the discovery of an extremely potent THC-like phytocannabinoid may shed light on several pharmacological effects not ascribable solely to Δ^9^-THC.

Ongoing studies are devoted to the investigation of the pharmacological activity of CBDP and to expand that of Δ^9^-THCP. It is known that CBD binds with poor affinity to both CB_1_ and CB_2_ receptors^55^. Therefore, the evaluation of the cannabimimetic activity of CBDP does not appear to be a high priority, although science can hold great surprises. Our current work is rather focused on testing its anti-inflammatory, anti-oxidant and anti-epileptic activity, which are typical of CBD^46^.

## Methods

### Plant material

FM2 cannabis variety is obtained from the strain CIN-RO produced by the Council for Agricultural Research and Economics (CREA) in Rovigo (Italy) and provided to the Military Chemical Pharmaceutical Institute (MCPI, Firenze, Italy) for breeding. FM2 inflorescence (batch n. 6A32/1) was supplied by the MCPI with the authorization of the Italian Ministry of Health (prot. n. SP/062). The raw plant material (10 g) was finely grinded and divided into two batches: one batch (500 mg) was extracted with 50 mL of ethanol 96% according to the procedure indicated by the monograph of *Cannabis Flos* of the German Pharmacopoeia^56^ and was analyzed by UHPLC-HESI-Orbitrap after proper dilution with acetonitrile (×100). The remaining 9.5 g were extracted following the protocol of Pellati *et al*. with some modifications^26^. Briefly, freeze-dried plant material was extracted with 400 mL of *n*-hexane for 15 min under sonication in an ice bath. Samples were centrifuged for 10 min at 2000 × *g* and the supernatants collected. The procedure was repeated twice more on the pellets. The combined supernatants were then dried under reduced pressure and resuspended in 10 mL of acetonitrile, filtered and used for the isolation of CBDPA and THCPA by semi-preparative liquid chromatography.

### Isolation of natural CBDP and Δ^9^-THCP

Aliquots (1 mL) of the solution obtained as described in the ‘Plant Material’ section were injected in a semi-preparative LC system (Octave 10 Semba Bioscience, Madison, USA). The chromatographic conditions used are reported in the paper by Citti *et al*.^11^. The column employed was a Luna C_18_ with a fully porous silica stationary phase (Luna 5 µm C18(2) 100 Å, 250 × 10 mm) (Phenomenex, Bologna, Italy) and a mixture of acetronitrile:0.1% aqueous formic acid 70:30 (v/v) was used as mobile phase at a flow rate of 5 mL/min. CBDPA and THCPA (retention time 19.0 min and 75.5 min respectively) were isolated as reported in our previous work^11^. The fractions containing CBDPA and THCPA were analyzed by UHPLC-HESI-Orbitrap. The fractions containing predominantly either one or the other cannabinoid were separately combined and dried on the rotavapor at 70 °C. Each residue was subject to decarboxylation at 120 °C for two hours in oven. An amount of about 0.6 mg of CBDP and about 0.3 mg of Δ^9^-THCP was obtained.

### UHPLC-HESI-Orbitrap metabolomic analysis

FM2 extracts were analyzed on a Thermo Fisher Scientific Ultimate 3000 system equipped with a vacuum degasser, a binary pump, a thermostated autosampler, a thermostated column compartment and interfaced to a heated electrospray ionization source and a Q-Exactive Orbitrap mass spectrometer (UHPLC-HESI-Orbitrap). The parameters of the HESI source were set according to Citti *et al*.^11^: capillary temperature, 320 °C; vaporizer temperature, 280 °C; electrospray voltage, 4.2 kV (positive mode) and 3.8 kV (negative mode); sheath gas, 55 arbitrary units; auxiliary gas, 30 arbitrary units; S lens RF level, 45. Analyses were acquired using the Xcalibur 3.0 software (Thermo Fisher Scientific, San Jose, CA, USA) in full scan data-dependent acquisition (FS-dd-MS^2^) in positive (ESI+) and negative (ESI−) mode at a resolving power of 70,000 FWHM at *m/z* 200. A scan range of *m/z* 250–400, an AGC of 3e6, an injection time of 100 ms and an isolation window for the filtration of the precursor ions of *m/z* 0.7 were chosen as the optimal parameters for the mass analyzer. A normalized collision energy (NCE) of 20 was used to fragment the precursor ions. Extracted ion chromatograms (EIC) of the [M + H]^+^ and [M-H]^−^ molecular ions were derived from the total ion chromatogram (TIC) of the FM2 extracts and matched with pure analytical standards for accuracy of the exact mass (5 ppm), retention time and MS/MS spectrum.

The chromatographic separation was carried out on a Poroshell 120 SB-C18 (3.0 × 100 mm, 2.7 µm, Agilent, Milan, Italy) following the conditions employed for our previous work^11^.

A semi-quantitative analysis of Δ^9^-THC and CBD and their heptyl analogs CBDP and Δ^9^-THCP was achieved using a calibration curve with an external standard. A stock solution of CBD and Δ^9^-THC, CBDP and Δ^9^-THCP (1 mg/mL) was properly diluted to obtain five non-zero calibration points at the final concentrations of 50, 100, 250, 500, and 1000 ng/mL for CBD and Δ^9^-THC and of 1, 5, 10, 25, and 50 ng/mL for CBDP and Δ^9^-THCP. A standard solution of Δ^9^-THC-*d*_3_ was added at each calibration standard at a final concentration of 50 ng/mL. The linearity was assessed by the coefficient of determination (*R*^2^), which was greater than 0.993 for each analyte.

### Synthetic procedure

All reagents and solvents were employed as purchased without further purification unless otherwise specified. The following abbreviations for common organic solvents have been used herein: diethyl ether (Et_2_O); dichloromethane (DCM); cyclohexane (CE). Reaction monitoring was performed by thin-layer chromatography on silica gel (60F-254, E. Merck) and checked by UV light, or alkaline KMnO_4_ aqueous solution^57–59^. Reaction products were purified, when necessary, by flash chromatography on silica gel (40−63 μm) with the solvent system indicated. NMR spectra were recorded on a Bruker 400 or Bruker 600 spectrometer working respectively at 400.134 MHz and 600.130 MHz for ^1^H and at 100.62 MHz or 150.902 MHz for ^13^C. Chemical shifts (δ) are in parts per million (ppm) and they were referenced to the solvent residual peaks (CDCl_3_ δ = 7.26 ppm for proton and δ = 77.20 ppm for carbon); coupling constants are reported in hertz (Hz); splitting patterns are expressed with the following abbreviations: singlet (s), doublet (d), triplet (t), quartet (q), double doublet (dd), quintet (qnt), multiplet (m), broad signal (b). Monodimensional spectra were acquired with a spectral width of 8278 Hz (for ^1^H-NMR) and 23.9 kHz (for ^13^C-NMR), a relaxation delay of 1 s, and 32 and 1024 number of transients for ^1^H-NMR and ^13^C-NMR, respectively^12^. The COSY spectra were recorded as a 2048 × 256 matrix with 2 transients per t1 increment and processed as a 2048 × 1024 matrix; the HSQC spectra were collected as a 2048 × 256 matrix with 4 transients per t1 increment and processed as a 2048 × 1024 matrix, and the one-bond heteronuclear coupling value was set to 145 Hz; the HMBC spectra were collected as a 4096 × 256 matrix with 16 transients per t1 increment and processed as a 4096 × 1024 matrix, and the long-range coupling value was set to 8 Hz^12^. Circular dichroism (CD) and UV spectra were acquired on a Jasco (Tokyo, Japan) J-1100 spectropolarimeter using a 50 nm/min scanning speed. Quartz cells with a 10 mm path length were employed to record spectra in the 500–220 nm range^12^. Optical rotation (λ) was measured with a Polarimeter 241 (cell-length 100 mm, volume 1 mL) from Perkin-Elmer (Milan, Italy). The synthetic procedures described below were adjusted from previously published works^12,57^.

*Synthesis of (1*′*R,2*′*R)-4-heptyl-5*′*-methyl-2*′*-(prop-1-en-2-yl)-1*′*,2*′*,3*′*,4*′*-tetrahydro-[1,1*′*-biphenyl]-2,6-diol, (-)-trans-CBDP*

(1 *S*,4 *R*)-1-methyl-4-(prop-1-en-2-yl)cycloex-2-enol (146 mg, 0.96 mmol, 0.9 eq.), solubilized in 15 mL of anhydrous DCM, was added over a period of 20 min to a stirred solution of 5-heptylbenzene-1,3-diol (222 mg, 1.07 mmol, 1 eq.) and *p*-toluenesulfonic acid (20 mg, 0.11 mmol, 0.1 eq.) in anhydrous DCM (15 mL) at room temperature and under a positive pressure of argon. After stirring in the same conditions for 1 h, the reaction was quenched with 10 mL of a saturated aqueous solution of NaHCO_3_. The mixture was partitioned between Et_2_O and water. The organic layer was separated and washed with brine, dried with anhydrous Na_2_SO_4_ and evaporated. The residue was chromatographed (ratio crude:silica 1/120, eluent: CE:DCM 8/2). All the chromatographic fractions were analyzed by HPLC-UV and UHPLC-HESI-Orbitrap and only the fractions containing exclusively CBDP were concentrated to give 76 mg of a colorless oil (23% yield, purity > 99%).

^1^H NMR (400 MHz, CDCl_3_) δ 6.10–6.30 (m, 2 H), 5.97 (bs, 1 H), 5.57 (s, 1 H), 4.66 (s, 1 H), 4.66 (bs, 1 H), 4.56 (s, 1 H), 3.89–3.81 (m, 1 H), 2.52–2.35 (m, 3 H), 2.24 (td, *J* = 6.1, 12.7 Hz, 1 H), 2.09 (ddt, *J* = 2.4, 5.1, 17.9 Hz, 1 H), 1.89–1.74 (m, 5 H), 1.65 (s, 3 H), 1.55 (qnt, *J* = 7.6 Hz, 2 H), 1.28 (td, *J* = 4.7, 8.2, 9.0 Hz, 8 H), 0.87 (t, *J* = 6.7 Hz, 3 H). ^13^C NMR (101 MHz, CDCl_3_) δ 156.27, 154.09, 149.56, 143.23, 140.22, 124.30, 113.93, 111.01, 109.91, 108.26, 46.33, 37.46, 35.70, 31.99, 31.14, 30.59, 29.43, 29.35, 28.60, 23.86, 22.84, 20.71, 14.29. HRMS m/z [M + H]^+^ calcd. for C_23_H_35_O_2_^+^: 343.2632. Found: 343.2629; [M-H]^−^ calcd. for C_23_H_33_O_2_^−^: 341.2475. Found: 341.2482. [α]_D_^20^ = −146° (*c* 1.0, ACN).

*Synthesis of (6aR,10aR)-3-heptyl-6,6,9-trimethyl-6a,7,10,10a-tetrahydro-6H-benzo[c]chromen-1-ol, (-)-trans-Δ* ^8^*-THCP*

The set-up of the reaction for the synthesis of *(-)-trans-Δ*^8^*-THCP* was performed as described for *(-)-trans-CBDP* and the resulting mixture was stirred at room temperature for 48 h. The mixture was diluted with Et_2_O, and washed with a saturated solution of NaHCO_3_ (10 mL). The organic layer was collected, washed with brine, dried (anhydrous Na_2_SO_4_) and concentrated. After purification over silica gel (ratio crude:silica 1/150, eluent: CE:Et_2_O 95/5) 315 mg of a colorless oil (46% yield) were obtained.

^1^H NMR (400 MHz, CDCl_3_) δ 6.28 (d, *J* = 1.6 Hz, 1 H), 6.10 (d, *J* = 1.6 Hz, 1 H), 5.46–5.39 (m, 1 H), 4.78 (s, 1 H), 3.20 (dd, *J* = 4.5, 16.0 Hz, 1 H), 2.70 (td, *J* = 4.7, 10.8 Hz, 1 H), 2.44 (td, *J* = 2.3, 7.4 Hz, 2 H), 2.21–2.10 (m, 1 H), 1.92–1.76 (m, 3 H), 1.70 (s, 3 H), 1.63–1.52 (m, 2 H), 1.38 (s, 3 H), 1.30 (tt, *J* = 4.3, 9.4, 11.8 Hz, 8 H), 1.11 (s, 3 H), 0.88 (t, *J* = 7.0 Hz, 3 H).

*Synthesis of (6aR,10aR)-3-heptyl-*9*-chloro-6,6,9-trimethyl-6a,7,8,9,10,10a-hexahydro-6H-benzo[c]chromen-1-ol (HCl-THCP)*

1 N ZnCl_2_ in Et_2_O (440 µL, 0.44 mmol, 0.5 eq.) was added to a stirred solution of Δ^8^-THCP (300 mg, 0.87 mmol, 1 eq.) in 20 mL of anhydrous DCM, at room temperature and under nitrogen atmosphere. After 30 min, the reaction was cooled at 0 °C and 2 mL of 4 N HCl in dioxane was added. The resulting mixture was stirred at room temperature, overnight and then diluted with Et_2_O. The organic layer was collected and washed, in sequence, with an aqueous saturated solution of NaHCO_3_ and brine. After dehydration with anhydrous Na_2_SO_4_, the organic phase was concentrated to give 305 mg (93% yield) of a yellowish oil, pure enough to be used in the next step without further purification.

^1^H NMR (400 MHz, CDCl_3_) δ 6.24 (d, *J* = 1.7 Hz, 1 H), 6.07 (d, *J* = 1.6 Hz, 1 H), 4.94 (s, 1 H), 3.45 (dd, *J* = 2.9, 14.4 Hz, 1 H), 3.05 (td, *J* = 2.9, 11.3 Hz, 1 H), 2.42 (td, *J* = 1.5, 7.4 Hz, 2 H), 2.20–2.12 (m, 1 H), 1.80–1.71 (m, 1 H), 1.66 (s, 4 H), 1.60–1.51 (m, 2 H), 1.49–1.42 (m, 1 H), 1.38 (s, 3 H), 1.34–1.18 (m, 10 H), 1.13 (s, 3 H), 0.87 (t, *J* = 6.6 Hz, 3 H). ESI-MS m/z [M + H] + calcd. for C_23_H_36_^35^[Cl]O_2_^+^: 379.2. Found: 379.4. Calcd. for C_23_H_36_^37^[Cl]O_2_^+^: 381.2. Found: 381.3.

*Synthesis of (6aR,10aR)-3-heptyl-6,6,9-trimethyl-6a,7,8,10a-tetrahydro-6H-benzo[c]chromen-1-ol, (-)-trans-Δ*^*9*^*-THCP*.

HCl-THCP (305 mg, 0.82 mmol, 1 eq.) was solubilized in 10 mL of anhydrous toluene and cooled at −15 °C. 1.75 N potassium *t-*amylate in toluene (1.17 mL, 2.05 mmol, 2.5 eq.) was added dropwise with a syringe to the first solution under a positive pressure of argon. The mixture was stirred in the same condition for 15 min and then at 60 °C for 1 h. After cooling at room temperature, the reaction was quenched with a 1% solution of ascorbic acid and diluted with Et_2_O. The organic layer was washed with brine, dried over anhydrous Na_2_SO_4_ and concentrated. The residue was chromatographed (ratio crude/silica 1:300, hexane:*i*-propyl ether 9/1) to give 232 mg of a greenish oil (83% yield). 50 mg of (-)-*trans*-Δ^9^-THCP were further purified by semipreparative HPLC to prepare a pure analytic standard (purity > 99.9%).

^1^H NMR (600 MHz, CDCl_3_) δ 6.30 (t, *J* = 2.0 Hz, 1 H), 6.27 (d, *J* = 1.6 Hz, 1 H), 6.14 (d, *J* = 1.5 Hz, 1 H), 4.75 (s, 1 H), 3.20 (dt, *J* = 2.5, 10.8 Hz, 1 H), 2.43 (dd, *J* = 6.4, 8.9 Hz, 2 H), 2.22–2.11 (m, 2 H), 1.97–1.87 (m, 1 H), 1.69–1.65 (m, 4 H), 1.58–1.50 (m, 2 H), 1.43–1.37 (m, 4 H), 1.34–1.21 (m, 8 H), 1.09 (s, 3 H), 0.87 (t, *J* = 6.6 Hz, 3 H). ^13^C NMR (151 MHz, CDCl_3_) δ 154.97, 154.34, 143.02, 134.59, 123.92, 110.30, 109.22, 107.72, 77.38, 46.01, 35.72, 33.78, 31.99, 31.37, 31.16, 29.50, 29.38, 27.77, 25.22, 23.55, 22.87, 19.47, 14.29. HRMS m/z [M + H]^+^ calcd. for C_23_H_35_O_2_^+^: 343.2632. Found: 343.2633; [M-H]^−^ calcd. for C_23_H_33_O_2_^−^: 341.2475. Found: 341.2481. [α]_D_^20^ = −166° (*c* 1.0, ACN).

### Binding at CB_1_ and CB_2_ Receptors

The binding affinity of (-)-*trans*-Δ^9^-THCP against human CB_1_ and CB_2_ receptors was assessed by Eurofins Discovery using a radioligand binding assay. Ten concentrations of the phytocannabinoid from 1 nM to 30 µM were tested in duplicate. [^3^H]CP55940 (at 2 nM, *K*_*d*_ = 2.4 nM) and [^3^H]WIN 55212-2 (at 0.8 nM, *K*_*d*_ = 1.5 nM) were used as specific radioligand for *h*CB_1_ and *h*CB_2_, respectively^60,61^. Equation 1 was employed to calculate the percent inhibition (*%in*) of control specific binding obtained in the presence of the tested compounds.

1 $$ \% in=100-(\frac{measured\,specific\,binding}{control\,specific\,binding}\ast 100)$$

A non-linear regression analysis of the competition curves generated with mean replicate values (Eq. 2) was used to calculate the IC_50_ values (concentration causing a half-maximal inhibition of control specific binding)^62^.

2 $$Y=D+[\frac{A-D}{1+(\frac{C}{{C}_{50}})nH}]$$

Where Y is the specific binding, A is the left asymptote of the curve, D is the right asymptote f the curve, C is the compound concentration, C_50_ is the IC_50_ value and nH is the slope factor. This analysis was carried out using a software developed at Cerep (Hill software) and validated by comparing the data with that generated by the commercial software SigmaPlot 4.0 for Windows (1997 by SPSS Inc.). The inhibition constants (*K*_*i*_) were determined using the Cheng Prusoff equation (Eq. 3):

3 $$Ki=\frac{I{C}_{50}}{(1+\frac{L}{{K}_{D}})}$$

where L is the concentration of the radioligand, and *K*_*D*_ is the affinity of the radioligand for the receptor.

The data obtained for CP 55940 (CB_1_ IC_50_ = 1.7 nM, CB_1_ *K*_*i*_ = 0.93 nM) and WIN 55212-2 (CB_2_ IC_50_ = 2.7 nM, CB_2_ *K*_*i*_ = 1.7 nM) were in accordance with the values reported in literature^60,61^.

### Docking simulation

The prediction of the binding mode of Δ^9^-THCP in complex with human CB_1_ receptor was performed using Maestro 10.3 of the Schrödinger Suite^63^. The crystallographic structure of the active conformation of CB_1_ in complex with AM11542 (PDB ID: 5XRA) was downloaded from the Protein Data Bank and was used as reference for docking calculation. The protein was prepared using the Protein Preparation Wizard module^64^. The chemical structure of (-)-*trans*-Δ^9^-THCP was sketched with ChemDraw 12.0 and converted from 2D to 3D with the LigPrep utility^65^. Five conformations per ligand were initially generated, and appropriate ionization state and tautomers were evaluated for each conformation at physiological pH^66,67^. Afterwards, ligand conformations were minimized with the OPLS_2005 force field. Rigid docking was performed in extra precision mode with Glide version 6.8^68^.

### Tetrad test

Male C57BL6/J mice (7 weeks old; *n* = 5) were treated with Δ^9^-THCP (10, 5 and 2.5 mg/kg) or vehicle (1:1:18; ethanol:Kolliphor EL:0.9% saline) by i.p. administration. Mice were evaluated for hypomotility (open field test), hypothermia (body temperature), antinociceptive (hot plate test), and cataleptic (bar test) effects, using the procedures of the tetrad tests as reported by Metna-Laurent *et al*.^69^. The same animals were used in all four behavioral tests. Statistical analysis was performed using the Kruskall-Wallis test and Dunn’s post hoc tests.

### Body temperature

The mouse was immobilized and the probe gently inserted for 1 cm into the rectum until stabilization of temperature. Between each mouse the probe was cleaned with 70% ethanol and dried with paper towel.

### Open field

The open field test was used for the evaluation of motor activity. Behavioral assays were performed 30 min after drug (or vehicle) injection. The apparatus was cleaned before each behavioral session by a 70% ethanol solution. Naϊve mice were randomly assigned to a treatment group. Behaviors were recorded, stored, and analyzed using an automated behavioral tracking system (Smart v3.0, Panlab Harvard Apparatus). Mice were placed in an OFT arena (l × w × h: 44 cm × 44 cm × 30 cm), and ambulatory activity (total distance travelled in centimeter) was recorded for 15 min and analyzed.

### Bar test

The bar test was used for the evaluation of catalepsy. The bar was a 40 cm in length and 0.4 cm in diameter glass rod, which was horizontally elevated by 5 cm above the surface. Both forelimbs of the mouse were positioned on the bar and its hind legs on the floor of the cage, ensuring that the mouse was not lying down on the floor. The chronometer was stopped when the mouse descended from the bar (i.e., when the two forepaws touched the floor) or when 10 min had elapsed (i.e., cut-off time). Catalepsy was measured as the time duration each mouse held the elevated bar by both its forelimbs (latency for moving in seconds).

### Hot plate

The hot plate test was performed to assess changes in the nociception. On the day of the experiment each mouse was placed on a hot plate (Ugo Basile) that was kept at a constant temperature of 52 °C. Licking of the hind paws or jumping were considered as a nociceptive response (NR) and the latency was measured in seconds 85 minutes after drug or vehicle administration, taking a cut-off time of 30 or 60 s in order to prevent tissue damage.

### Animals

Male C57BL/6 mice (Charles River, Italy) of 18–20 g weight were used for the tetrad experiments. A 12 h light/dark cycle with light on at 6:00 A.M., a constant temperature of 20–22 °C, and a 55–60% humidity were maintained for at least 1 week before beginning the experiments. Mice were housed three per cage with chow and tap water available *ad libitum*. The experimental procedures employed for the work presented herein were approved by the Animal Ethics Committee of the University of Campania “L. Vanvitelli”, Naples. Animal care and welfare were entrusted to adequately trained personnel in compliance with Italian (D.L. 116/92) and European Commission (O.J. of E.C. L358/1, 18/12/86) regulations on the protection of animals used for research purposes. All efforts were made to minimize animal numbers and avoid unnecessary suffering during the experiments.

## Supplementary information

Supplementary Information.
