# Supplementary material for: A novel phytocannabinoid isolated from Cannabis sativa L. with an in vivo cannabimimetic activity higher than Δ9-tetrahydrocannabinol: Δ9-Tetrahydrocannabiphorol
Source: Sci Rep. 2019 Dec 30;9:20335. doi: 10.1038/s41598-019-56785-1 (PMC6937300; doi:10.1038/s41598-019-56785-1)
Supplement: Supplementary file 1 — Supplementary Information. [file 41598_2019_56785_MOESM1_ESM.docx]

**A novel phytocannabinoid isolated from *Cannabis sativa* L. with an *in vivo* cannabimimetic activity higher than Δ^9^-tetrahydrocannabinol: Δ^9^-Tetrahydrocannabiphorol**

Cinzia Citti^1,2,†^, Pasquale Linciano^3,†^, Fabiana Russo^3^, Livio Luongo^4^, Monica Iannotta^4^, Sabatino Maione^4^, Aldo Laganà^2,5^, Anna Laura Capriotti^5^, Flavio Forni^3^, Maria Angela Vandelli^3^, Giuseppe Gigli^2^, Giuseppe Cannazza^2,3,*^

1. Mediteknology (CNR spin-off company), Via Arnesano, 73100 Lecce
2. CNR NANOTEC, Istituto di Nanotecnologia, Via Monteroni, 73100 Lecce, Italy
3. Department of Life Sciences, University of Modena and Reggio Emilia, Via Campi 103, 41125 Modena, Italy
4. Department of Experimental Medicine, Division of Pharmacology, Università della Campania “L. Vanvitelli”, Via Santa Maria di Costantinopoli 16, 80138 Naples, Italy
5. Department of Chemistry, Sapienza University of Rome, Piazzale Aldo Moro 5, 00185 Rome, Italy

^†^ These authors contributed equally to the work

- Corresponding author: [giuseppe.cannazza@unimore.it](mailto:giuseppe.cannazza@unimore.it), tel: +39 059 2055013, fax: +39 059 2055750, ORCID ID: 0000-0002-7347-7315

**Table of Content**

| **Procedure for the synthesis of 5-heptylbenzene-1,3-diol (1)** | … SI-2 |
| --- | --- |
| **Table SI-1.** ^1^H and ^13^ NMR assignments (δ) of (-)-*trans*-CBDP | … SI-4 |
| **Table SI-2.** ^1^H and ^13^ NMR assignments (δ) of (-)-*trans*-Δ^9^-THCP | … SI-5 |
| **Figure SI-1.** Synthetic procedure for the synthesis of sphaerophorol (1) | … SI-6 |
| **Figure SI-2.** NMR spectroscopic characterization of synthetic (-)-*trans*-CBDP | … SI-7 |
| **Figure SI-3.** NMR spectroscopic characterization of synthetic (-)-*trans*-Δ^9^-THCP | … SI-10 |
| **Figure SI-4.** NMR spectroscopic characterization of extracted (-)-*trans*-CBDP | … SI-13 |
| **Figure SI-5.** NMR spectroscopic characterization of extracted (-)-*trans*-Δ^9^-THCP | … SI-14 |
| **Figure SI-6.** Crystal structure of hCB1 in complex with AM11542 agonist | … SI-15 |
| **Figure SI-7.** Predicted binding mode THC, THCV, THCB | … SI-16 |

**Procedure for the synthesis of 5-heptylbenzene-1,3-diol (1).** The synthesis of 5-heptylbenzene-1,3-diol, also known as 5-heptyl-resorcinol or sphaerophorol is reported in Scheme S1. 1-(bromomethyl)-3,5-dimethoxybenzene was reacted first with triphenylphosphine in refluxing toluene for 6 hours to quantitatively give the respective phosphonium salt (**4**). **4** was reacted with hexanal by Wittig’s reaction in 0.1 M K_2_CO_3_ aqueous solution at reflux for 24 hours to give the alkene (**3**) as a 38:62 *cis:trans* mixture. The mixture of *cis:trans* alkene **3** was reduced by hydrogenation over palladium/charcoal, using the ThalesNano H-Cube flow reactor, to give the alkane **2** in quantitative yield. Finally, the demethylation of the two phenolic moieties of **2**, using BBr_3_ in anhydrous DCM, overnight at room temperature and under nitrogen atmosphere, gave 5-heptyl-resorcinol (**1**) in quantitative yield.

*Synthesis of (3,5-dimethoxybenzyl)triphenylphosphonium bromide (4)*

Triphenylphosphine (6.3 g, 23.8 mmol, 1.1 eq.) was added to a stirred solution of 1-(bromomethyl)-3,5-dimethoxybenzene (5.0 g, 21.6 mmol, 1 eq.), in 30 mL of toluene and refluxed for 6 hours. After standing at room temperature overnight, the precipitate formed was collected by filtration, washed with diethyl ether and dried to give 10.5 g of a white solid (quant. yield).

^1^H-NMR (400 MHz, CDCl_3_) δ 7.78-7.76 (m, 9H), 7.66-7.63 (m, 6H), 6.35 (t, 2H, *J* = 2.3 Hz), 6.30 (q, 1H, *J* = 2.3 Hz), 5.32 (d, 2H, *J* = 14.3 Hz), 3.54 (s, 6H).

*Synthesis of (E/Z)-1-(hept-1-en-1-yl)-3,5-dimethoxybenzene (3)*

**4** (5.0 g, 10 mmol, 1 eq.) was suspended in 100 mL of a 0.1 M solution of K_2_CO_3_ and hexanal (1.8 mL, 15 mmol, 1.5 eq.) was added in one portion. The suspension was refluxed for 24 hours and cooled at room temperature. 100 mL of cyclohexane was added, and the biphasic mixture was vigorously stirred at 0 °C for two hours. The precipitated triphenylphosphinoxide was removed by filtration. The organic phase was collected, and the aqueous phase was extracted two more times with cyclohexane. The combined organic phase was washed with brine, dried over anhydrous Na_2_SO_4_ and concentrated to give 2.325 g (99% yield) of a yellow oil. The product was obtained as a E/Z mixture of alkene, pure enough to be used in the next step without further purification.

^1^H NMR (400 MHz, CDCl_3_, Z-isomer) δ 6.43 (d, 2H, J = 2.2 Hz), 6.35 (t, 1H, J = 2.2 Hz), 6.33 (d, 1H, J = 11.5 Hz), 5.65 (dt, 1H, J = 11.5, J = 7.0 Hz), 3.97 (s, 6H), 2.32 (qd, 2H, J = 7.3 Hz, J = 1.7 Hz), 1.46 (quintet, 2H, J = 7.3 Hz), 1.40-1.21 (m, 4H), 0.88 (t, 3H, J = 7.1 Hz); ^1^H NMR (400 MHz, CDCl_3_, E-isomer) δ 6.50 (d, 2H, J = 2.2 Hz), 6.21 (dt, 1H, J = 15.8 Hz, J = 6.8 Hz), the remaining protons are overlapping with those of the Z-isomer.

*Synthesis of 1-heptyl-3,5-dimethoxybenzene (2)*

The mixture of (E/Z)-1-(hept-1-en-1-yl)-3,5-dimethoxybenzene (3) was solubilized in EtOH and the hydrogenation was performed with H-Cube Mini Plus ThalesNano using the following conditions: Temperature 30 °C, H_2_ 20 psi, cartridge Pd/C, solvent EtOH, flow 1mL/min. The solvent was concentrated to give 2.300g (98% yield) of a yellow liquid pure enough to be used in the next step without further purification.

^1^H NMR (400 MHz, CDCl_3_) δ 6.37 (d, *J* = 2.3 Hz, 2H), 6.32 (t, *J* = 2.3 Hz, 1H), 3.81 (s, 6H), 2.57 (dd, *J* = 6.8, 8.7 Hz, 2H), 1.72 – 1.59 (m, 2H), 1.41 – 1.22 (m, 8H), 0.91 (t, *J* = 6.8 Hz, 3H).

*Synthesis of 5-heptylbenzene-1,3-diol (1)*

To a solution of 2 (2.02 g, 8.62 mmol, 1 eq.) in anhydrous DCM at -10°C and under nitrogen atmosphere, a 1M solution of BBr3 in anhydrous DCM (19 mL, 19 mmol, 2.2 eq.) was added dropwise over a period of 30 minutes. The temperature was spontaneously risen, and the solution stirred at room temperature overnight. The reaction was quenched with an aqueous saturated solution of NaHCO_3_ and stirred until cessation of the effervescence. The organic phase was washed with brine, dried over anhydrous Na_2_SO_4_ and concentrated to give 1.780 g (99% yield) of a brown liquid which crystalized upon standing.

^1^H NMR (400 MHz, CDCl_3_) δ 6.24 (d, 2H, *J* = 2.3 Hz), 6.17 (t, 1H, *J* = 2.3 Hz), 2.48 (dd, 2H, *J* = 6.8, 8.7 Hz), 1.57 (t, 2H, *J* = 7.4 Hz), 1.39 – 1.19 (m, 8H), 0.88 (t, 3H, *J* = 7.4 Hz,).

**Table SI-1.** ^1^H and ^13^ NMR assignments (δ) of (-)-*trans*-CBDP

|  | | | |
| --- | --- | --- | --- |
| Position | ^1^H-NMR in CDCl_3_^a^ | | ^13^C-NMR in CDCl_3_^a^ |
| 1 | 3.89 – 3.81 (m, 1H) | | 37.46 |
| 2 | 5.57 (s, 1H) | | 124.30 |
| 3 | - | | 140.22 |
| 4 | a-2.09 (ddt, *J* = 2.4, 5.1, 17.9 Hz, 1H) | | 30.59 |
|  | b-2.24 (td, *J* = 6.1, 12.7 Hz, 1H) | |  |
| 5 | a,b-1.89 – 1.74 (m, 2H) | | 28.60 |
| 6 | 2.52 – 2.35 (m, 1H) | | 46.33 |
| 7 | 1.89 – 1.74 (m, 3H) | | 23.86 |
| 8 | - | | 149.56 |
| 9 | a-4.56 (s, 1H) | | 111.01 |
|  | b-4.66 (s, 1H) | |  |
| 10 | 1.65 (s, 3H) | | 20.71 |
| 1’ | - | | 154.09 |
| 2’ | - | | 113.93 |
| 3’ | - | | 156.27 |
| 4’ | 6.10-6.30 (m, 2H) | | 108.26 |
| 5’ | - | | 143.23 |
| 6’ | 6.10-6.30 (m, 2H) | | 109.91 |
| 7’ | 4.66 (bs, 1H) | | - |
| 8’ | 5.97 (bs, 1H) | | - |
| 1” | 2.52 – 2.35 (m, 3H) | | 35.70 |
| 2” | 1.55 (qnt, *J* = 7.6 Hz, 2H) | | 31.99 |
| 3” | } | 1.28 (td, *J* = 4.7, 8.2, 9.0 Hz, 8H) | 31.14 |
| 4” |  |  | 29.43 |
| 5” |  |  | 29.35 |
| 6” |  |  | 22.84 |
| 7” | 0.87 (t, *J* = 6.7 Hz, 3H) | | 14.29 |

^a^Chemical shifts are reported in parts per million (ppm, δ units). Proton chemical shifts were referenced to the solvent residual peak of CDCl_3_ (7.26 ppm). Splitting patterns are designed as s, singlet; t, triplet; sxt, sextet; dt, double triplet; m, multiplet; b, broad.

**Table SI-2.** ^1^H and ^13^ NMR assignments (δ) of (-)-*trans*-Δ^9^-THCP

|  | | | |
| --- | --- | --- | --- |
| Position | ^1^H-NMR in CDCl_3_^a^ | | ^13^C-NMR in CDCl_3_^a^ |
| 1 | 3.20 (dt, *J* = 2.5, 10.8 Hz, 1H) | | 33.78 |
| 2 | 6.30 (t, *J* = 2.0 Hz, 1H) | | 123.92 |
| 3 | - | | 134.59 |
| 4 | 2.22 – 2.11 (m, 2H) | | 31.37 |
| 5 | a- 1.97 – 1.87 (m, 1H)  b- 1.37-1.43 (m, 1H) | | 25.22 |
| 6 | 1.69-1.65 (m, 1H) | | 46.01 |
| 7 | - | | 77.38 |
| 8 | 1.41 (s, 3H) | | 27.77 |
| 9 | 1.09 (s, 3H) | | 19.47 |
| 10 | 1.68 (s, 3H) | | 23.55 |
| 1’ | - | | 110.30 |
| 2’ | - | | 154.97 |
| 3’ | 6.14 (d, *J* = 1.5 Hz, 1H) | | 107.72 |
| 4’ | - | | 143.02 |
| 5’ | 6.27 (d, *J* = 1.6 Hz, 1H) | | 109.22 |
| 6’ | - | | 154.34 |
| 7’ | 4.75 (bs, 1H) | | - |
| 1” | 2.43 (t, *J* = 8.9 Hz, 2H) | | 35.72 |
| 2” | 1.58 – 1.50 (m, 2H) | | 31.99 |
| 3” | } | 1.34 – 1.21 (m, 8H) | 31.16 |
| 4” |  |  | 29.50 |
| 5” |  |  | 29.38 |
| 6” |  |  | 22.87 |
| 7” | 0.87 (t, *J* = 6.6 Hz, 3H) | | 14.29 |

Chemical shift, in ppm, are referenced to the chloroform residual signal (7.26 ppm for ^1^H and 77.20 ppm for ^13^C). Coupling constants are reported in hertz (Hz). Splitting patterns are designed as s, singlet; d, doublet; t, triplet; qnt, quintet; sxt, sextet; m, multiplet; b, broad.

|  |
| --- |
| **Figure SI-1. Synthetic procedure for the synthesis of sphaerophorol (1).** Reagents and conditions: a) triphenylphosphine (1.1 eq.), toluene, reflux, 6h, quant. yield; b) hexanal (1.5 eq), 0.1 M K_2_CO_3_ aq (10 mL per mmol of **4**), reflux, 24h, 95% yield; c) H-Cube ThalesNano H_2_-Pd/C, EtOH, 30 °C, 20 bar, 1 mL / min, quant. yield; d) BBr_3_ 1M in DCM (2.2 eq.), anhydrous DCM, N_2_ atmosphere, -10°C 🡪 r.t, 24h, quant. yield. |

| **Figure SI-2.** Monodimensional (^1^H and ^13^C) and bidimensional (COSY, HSQC and HMBC) NMR spectroscopic characterization of synthetic (-)-*trans*-CBDP. |
| --- |
| 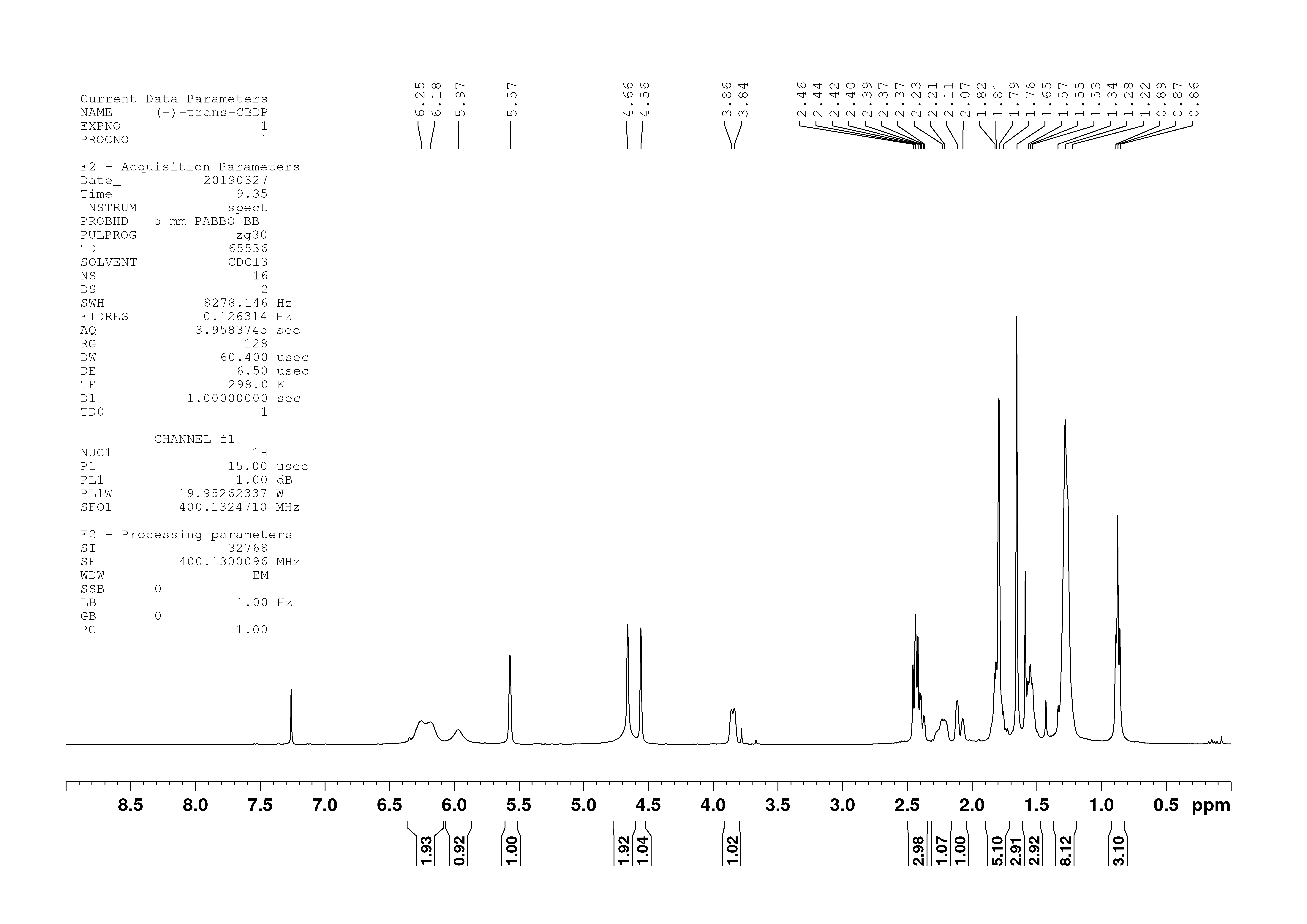 |
| 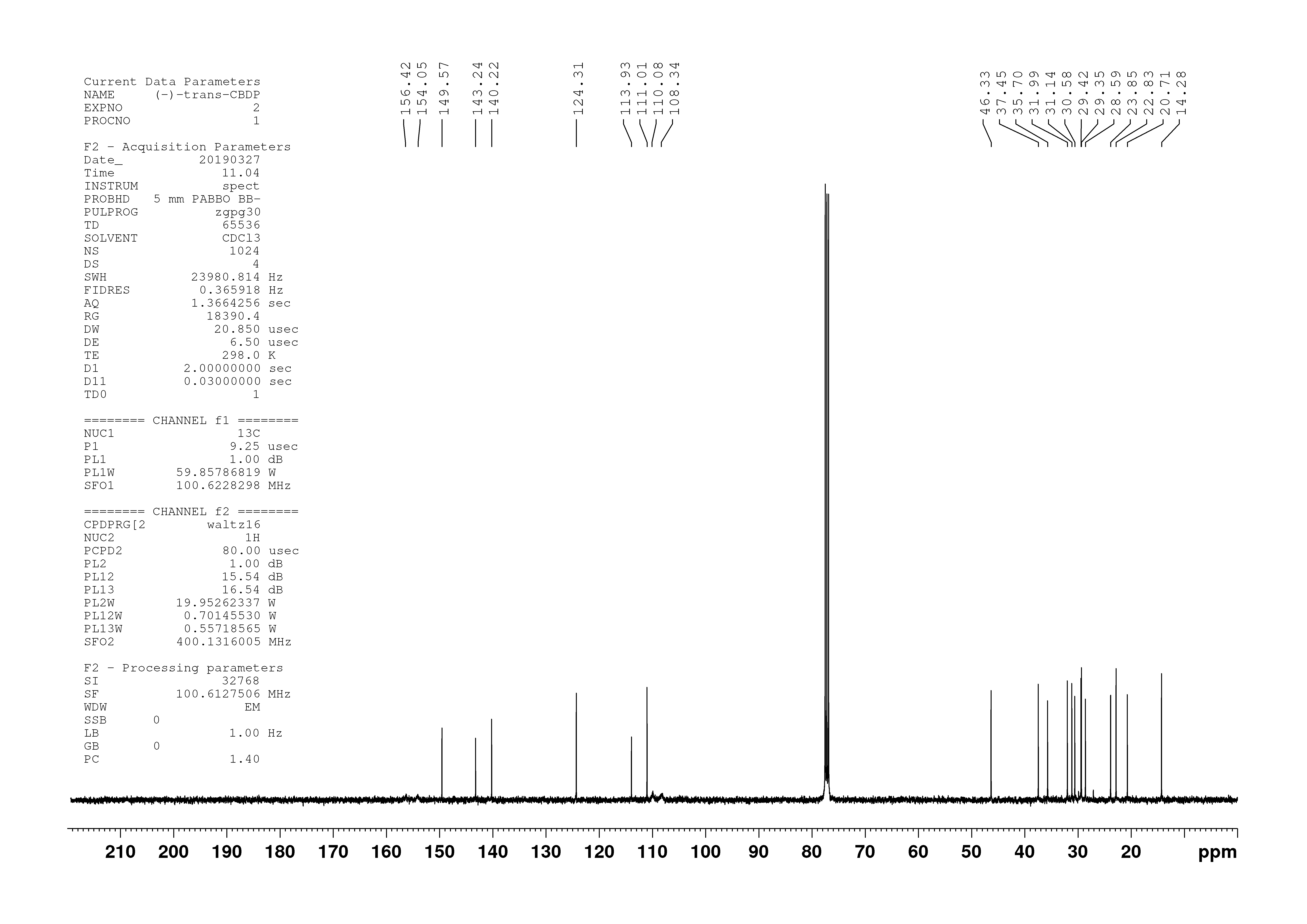 |
| 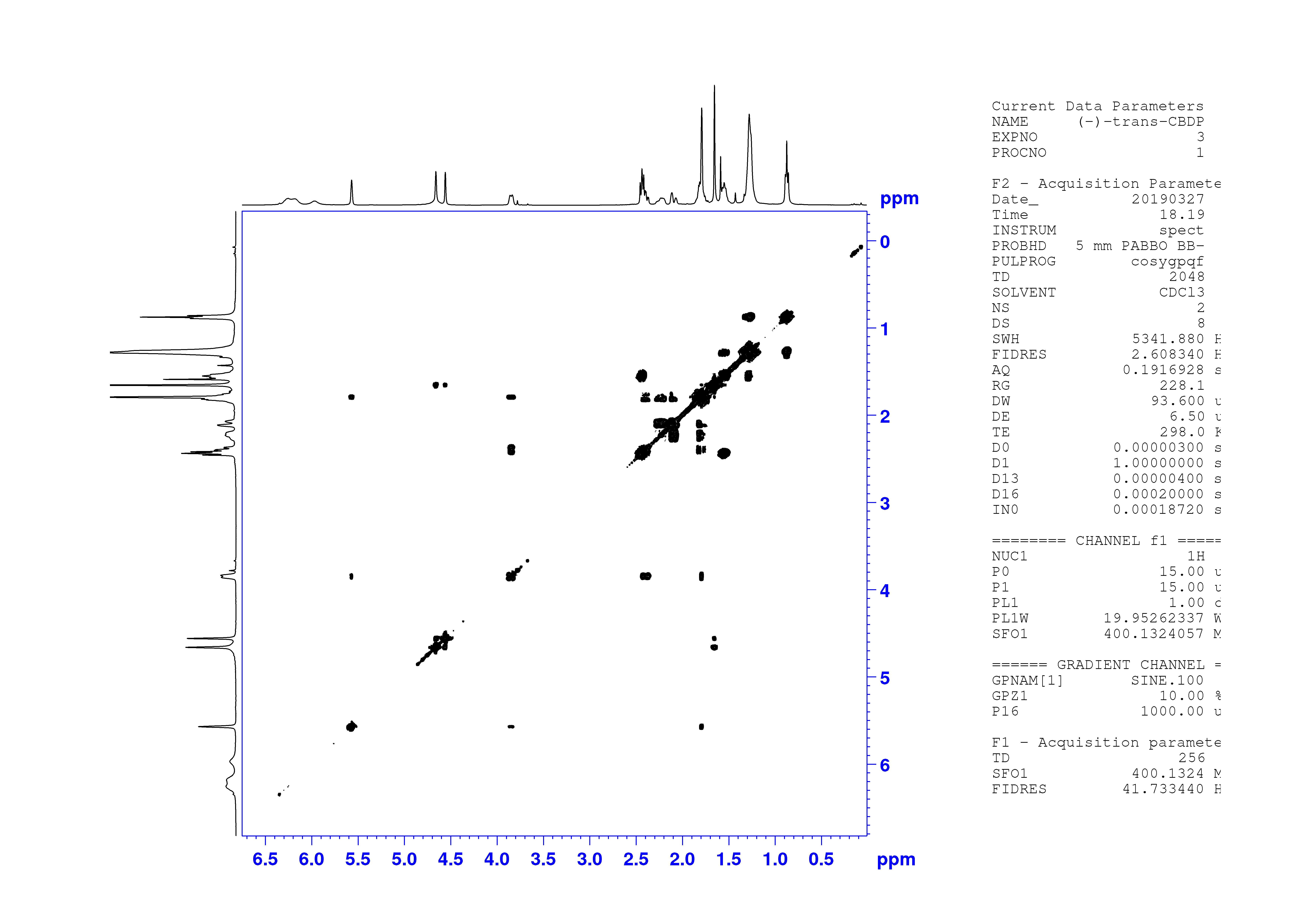 |
| 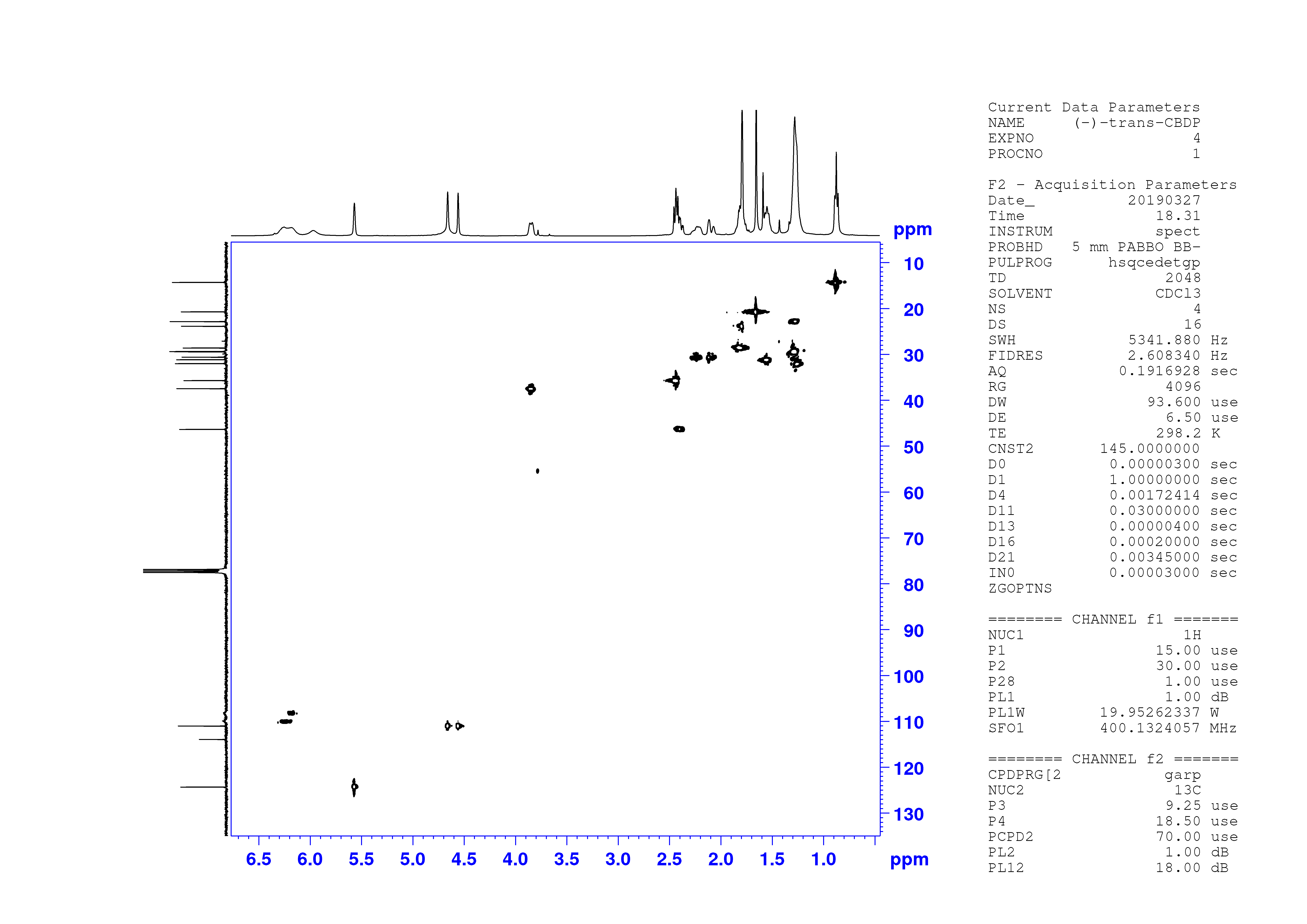 |
| 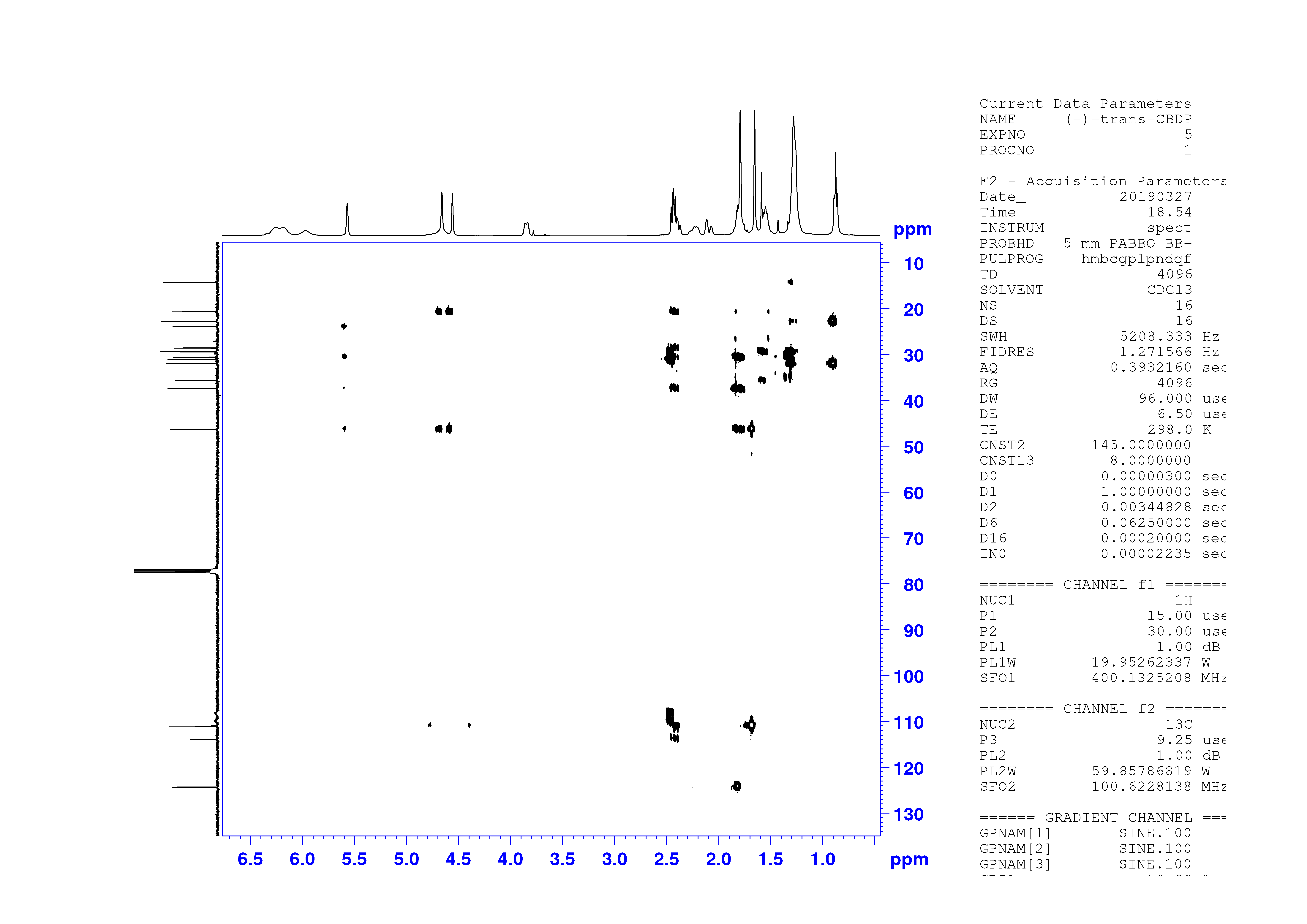 |

| **Figure SI-3.** Monodimensional (^1^H and ^13^C) and bidimensional (COSY, HSQC and HMBC) NMR spectroscopic characterization of synthetic (-)-*trans*-Δ^9^-THCP. |
| --- |
| 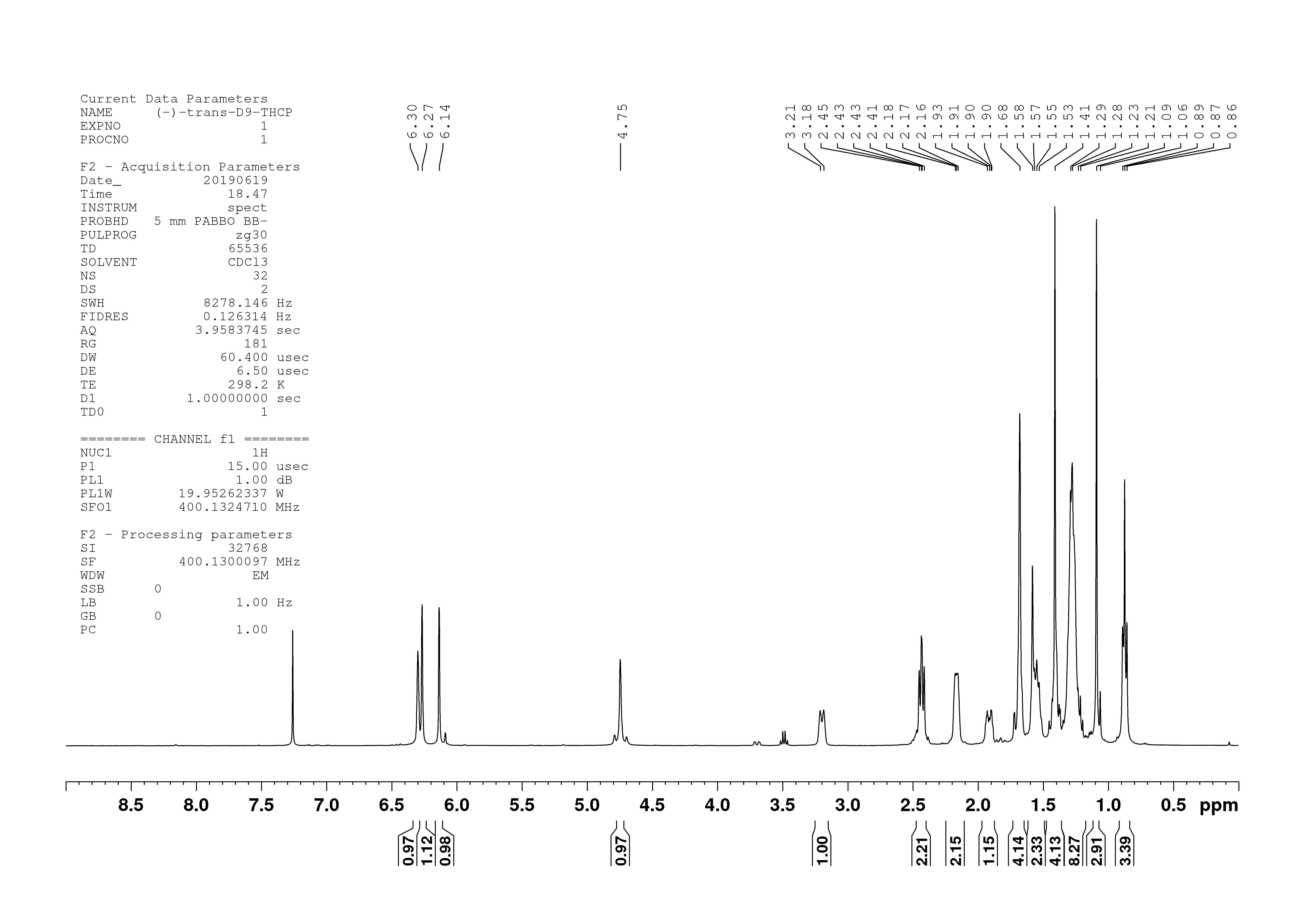 |
| 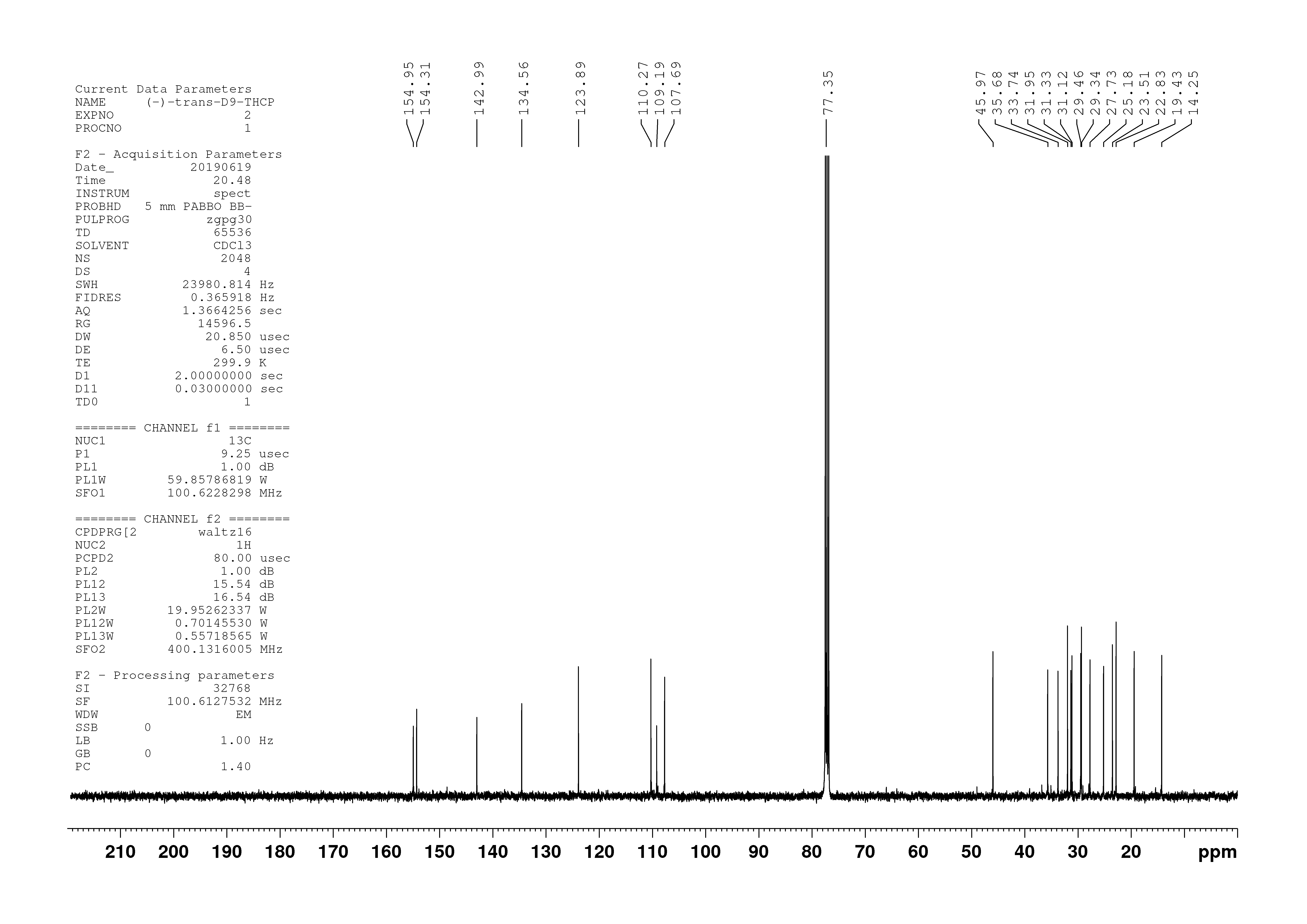 |
| 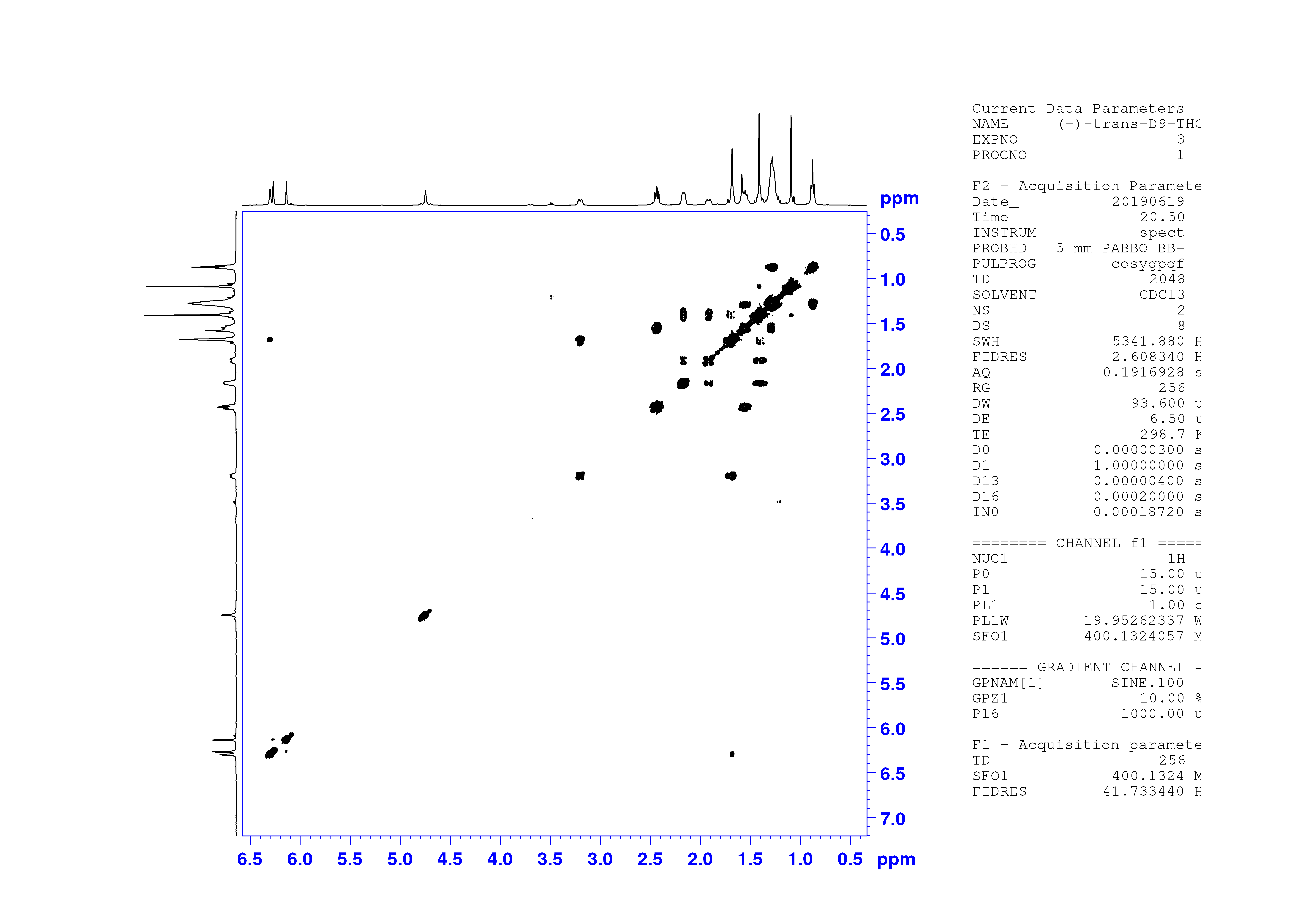 |
| 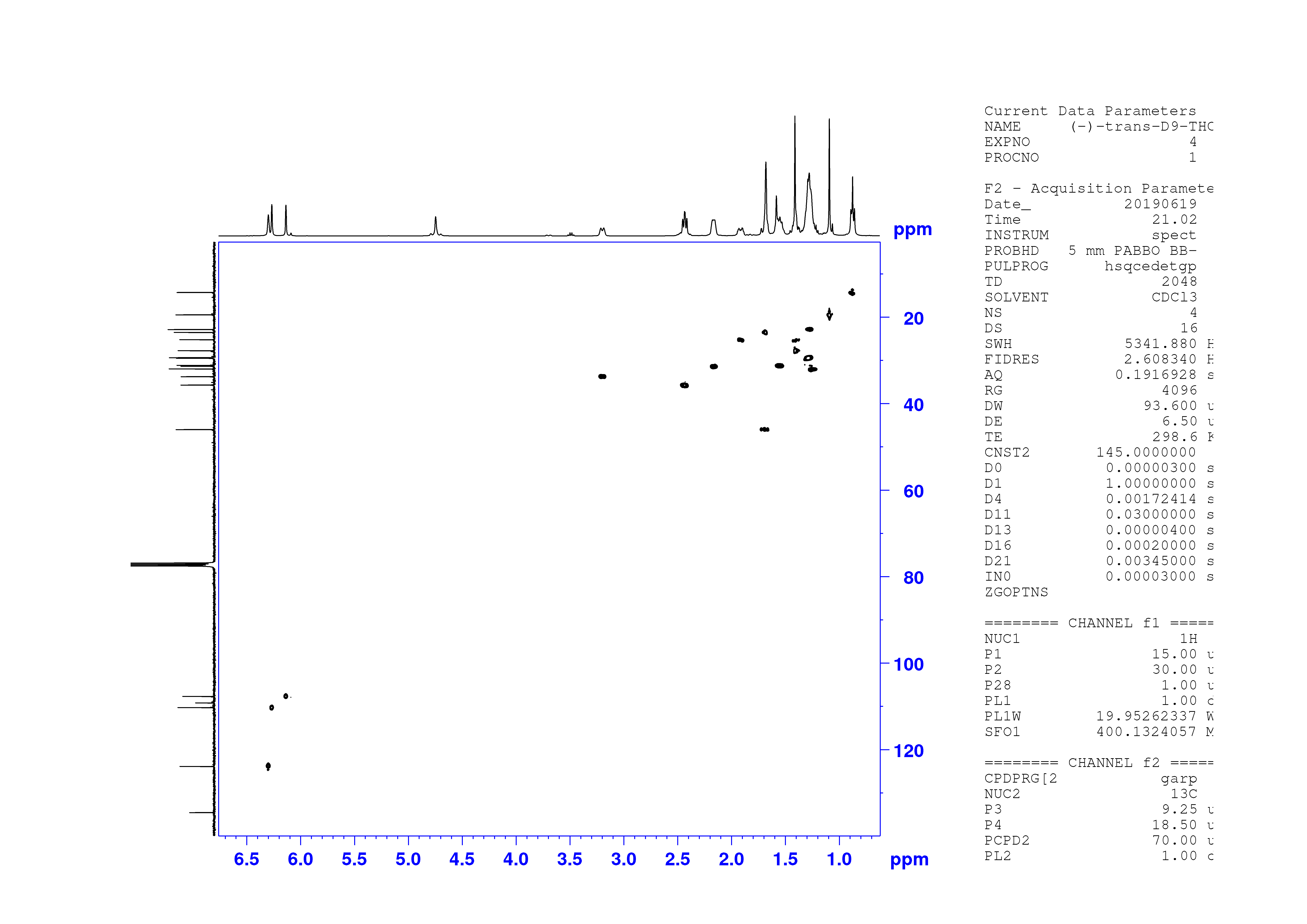 |
| 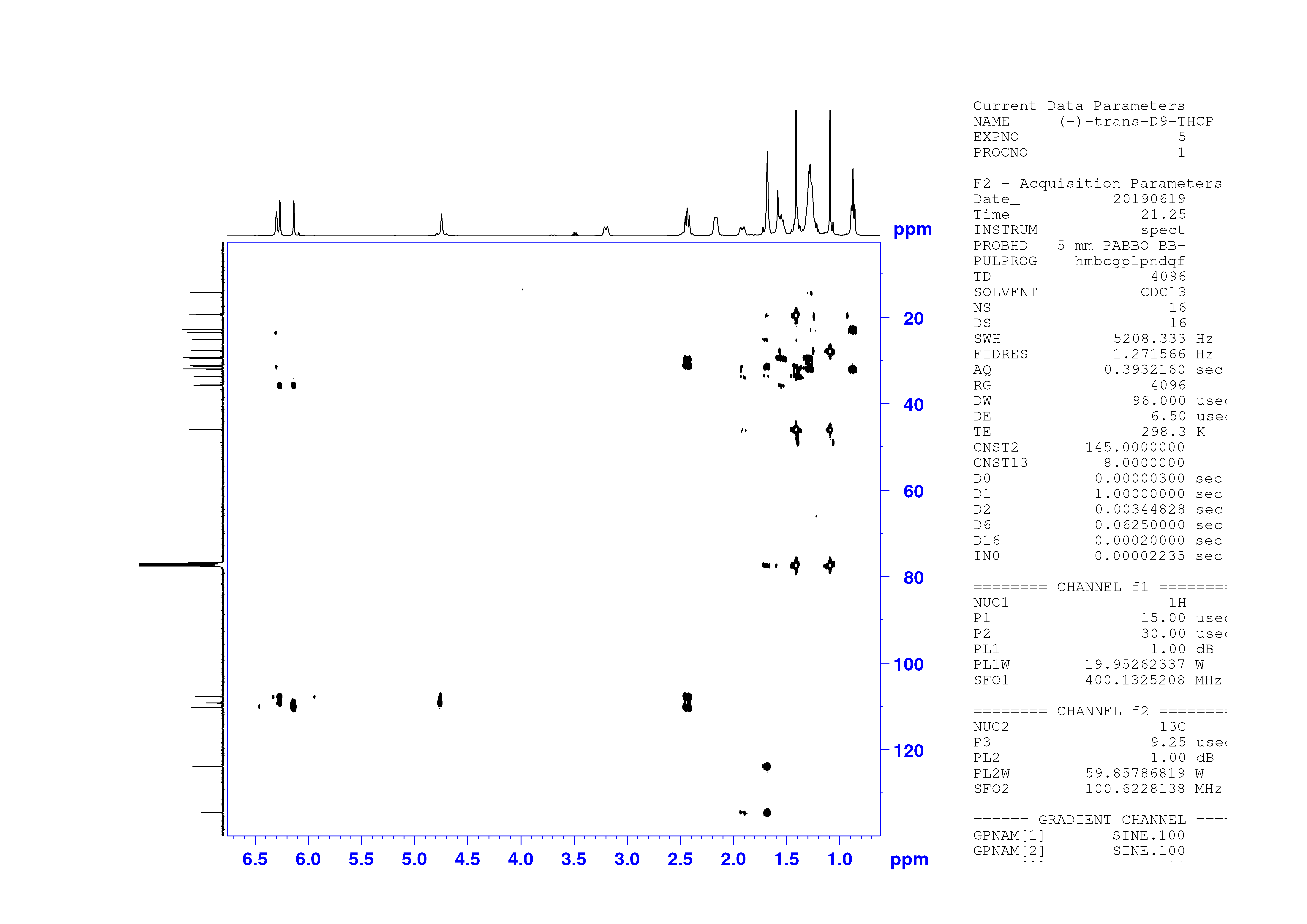 |

| **Figure SI-4.** ^1^H and ^13^C NMR spectra of extracted (-)-*trans*-CBDP |
| --- |
| 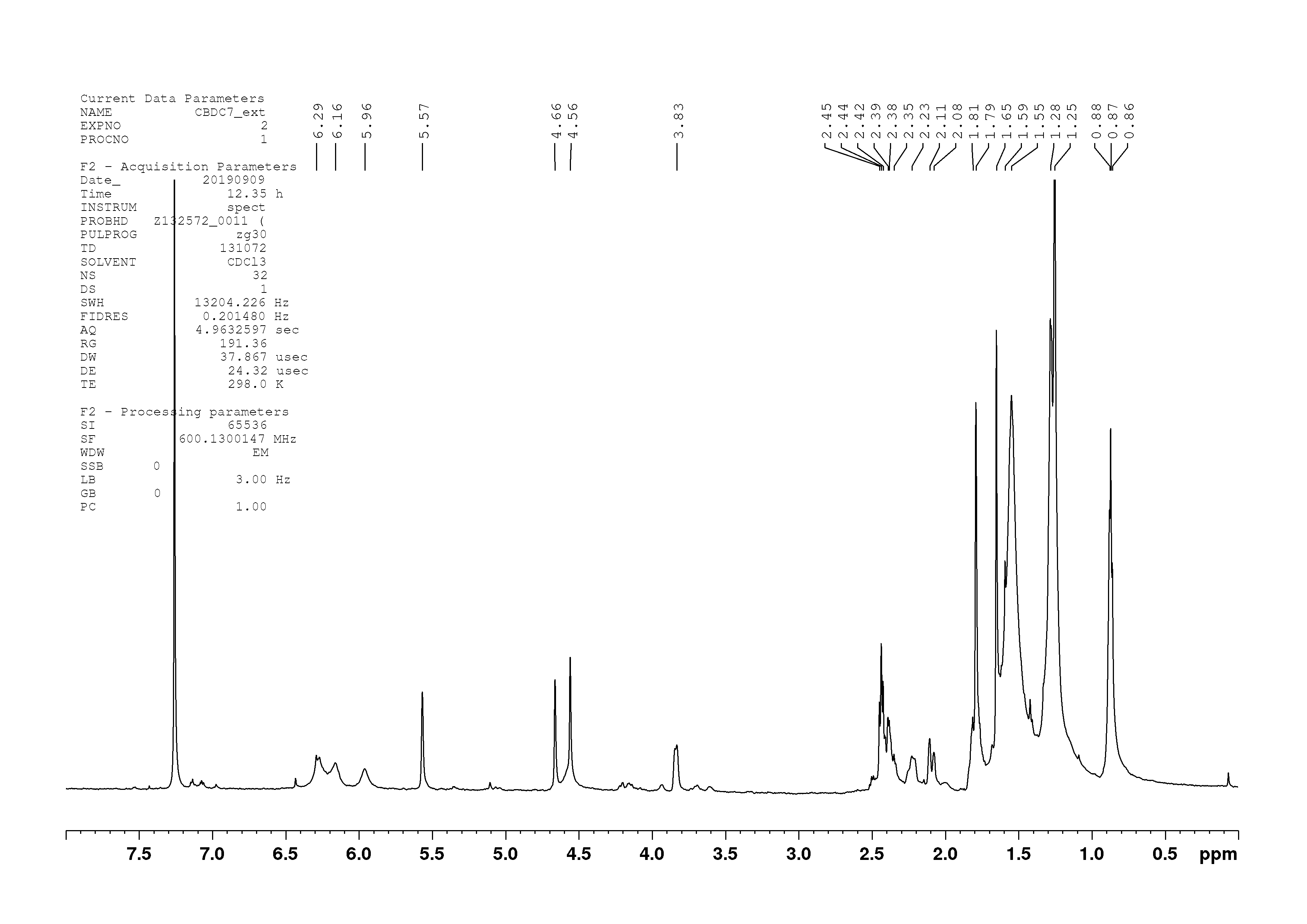 |
| 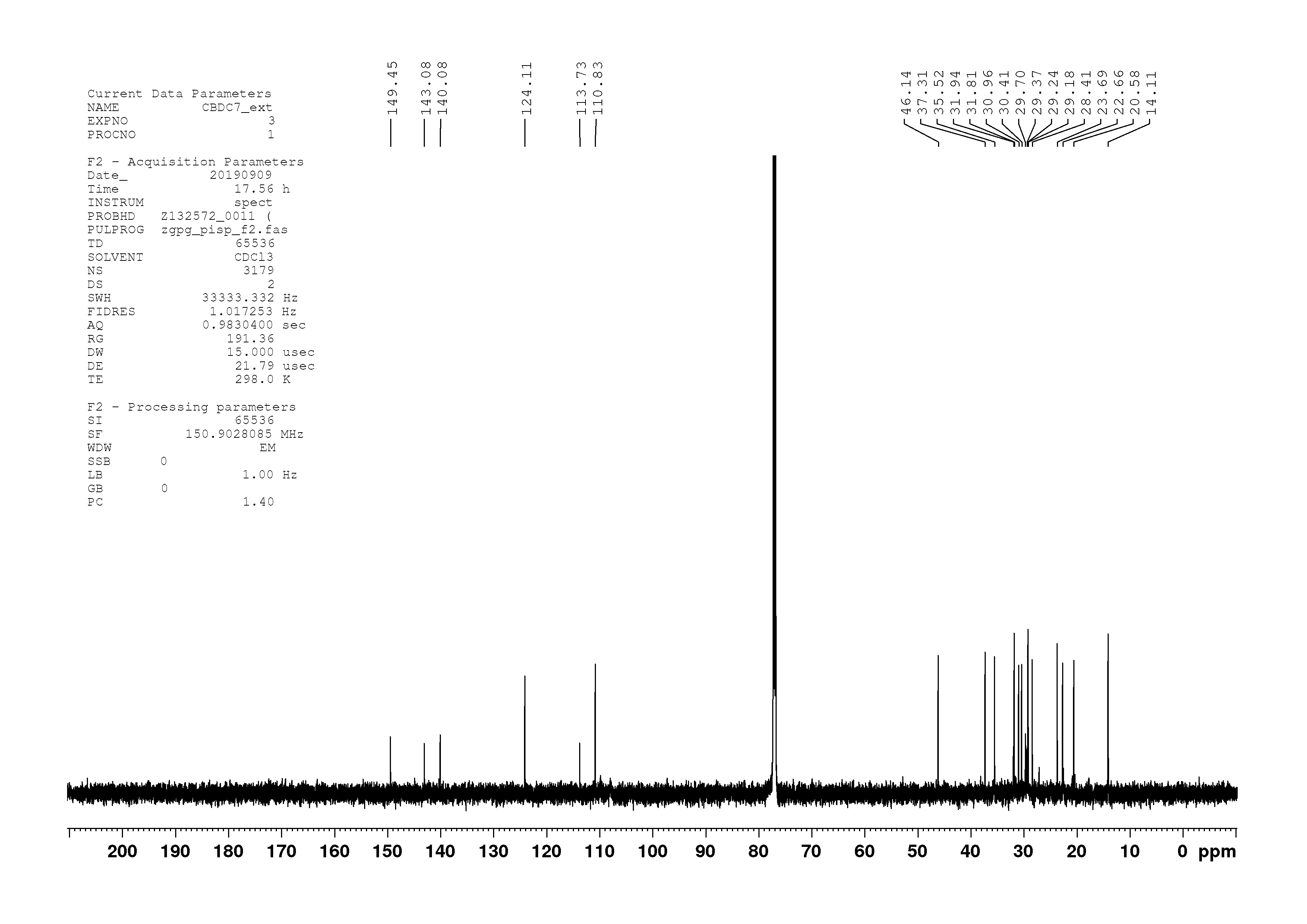 |

| **Figure SI-5.** ^1^H and ^13^C NMR spectra of extracted (-)-*trans*-Δ^9^-THCP |
| --- |
| 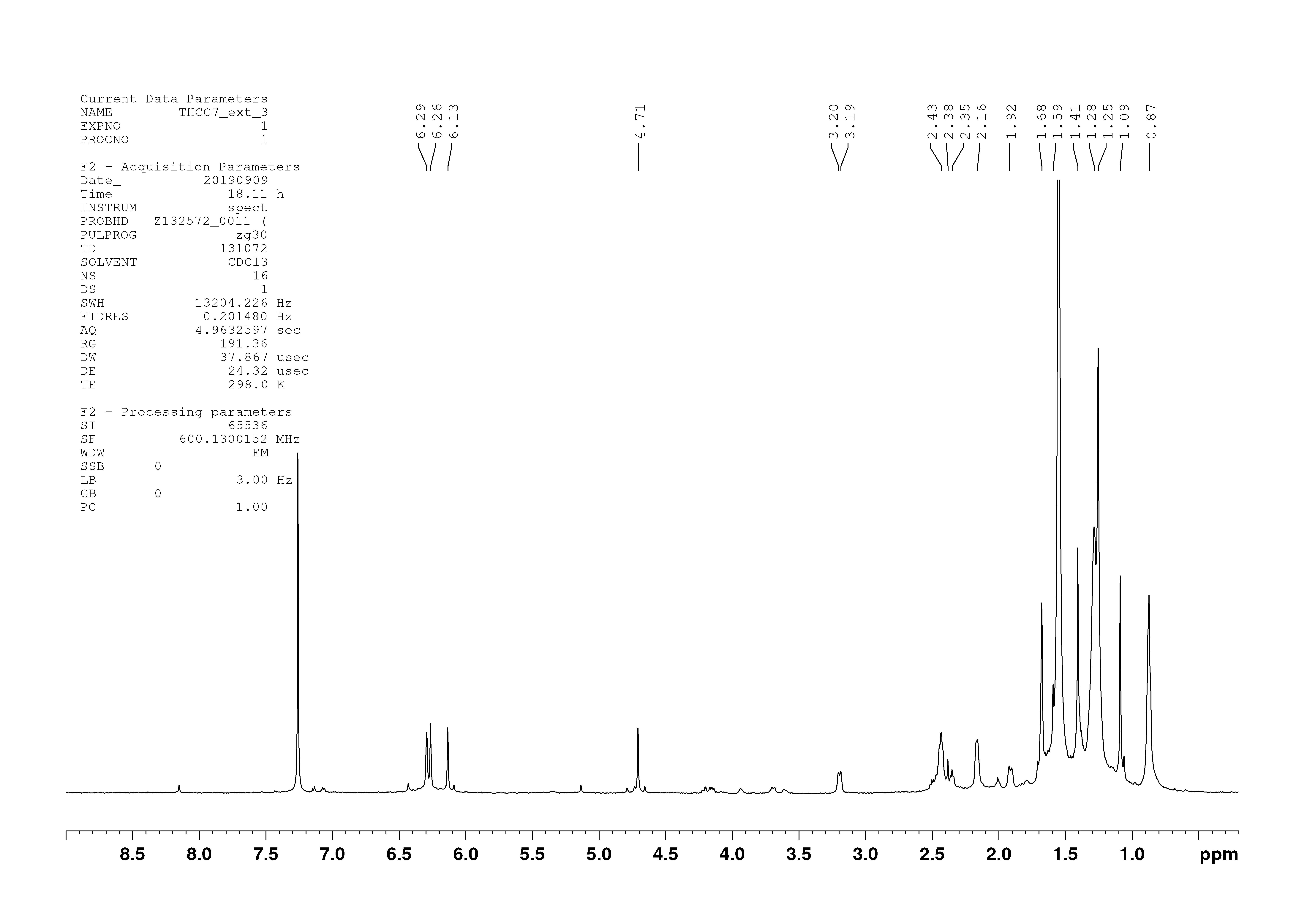 |
| 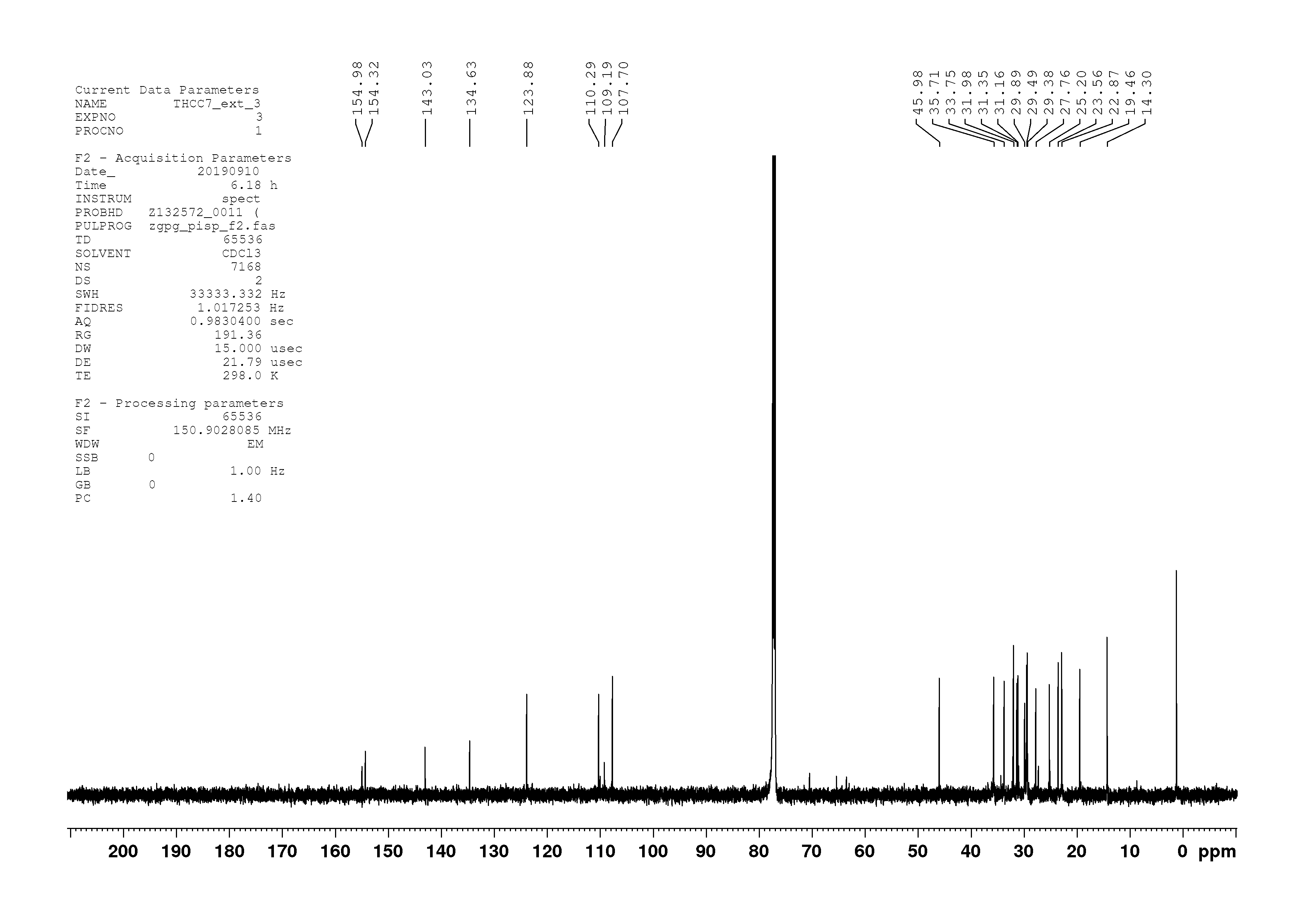 |

| 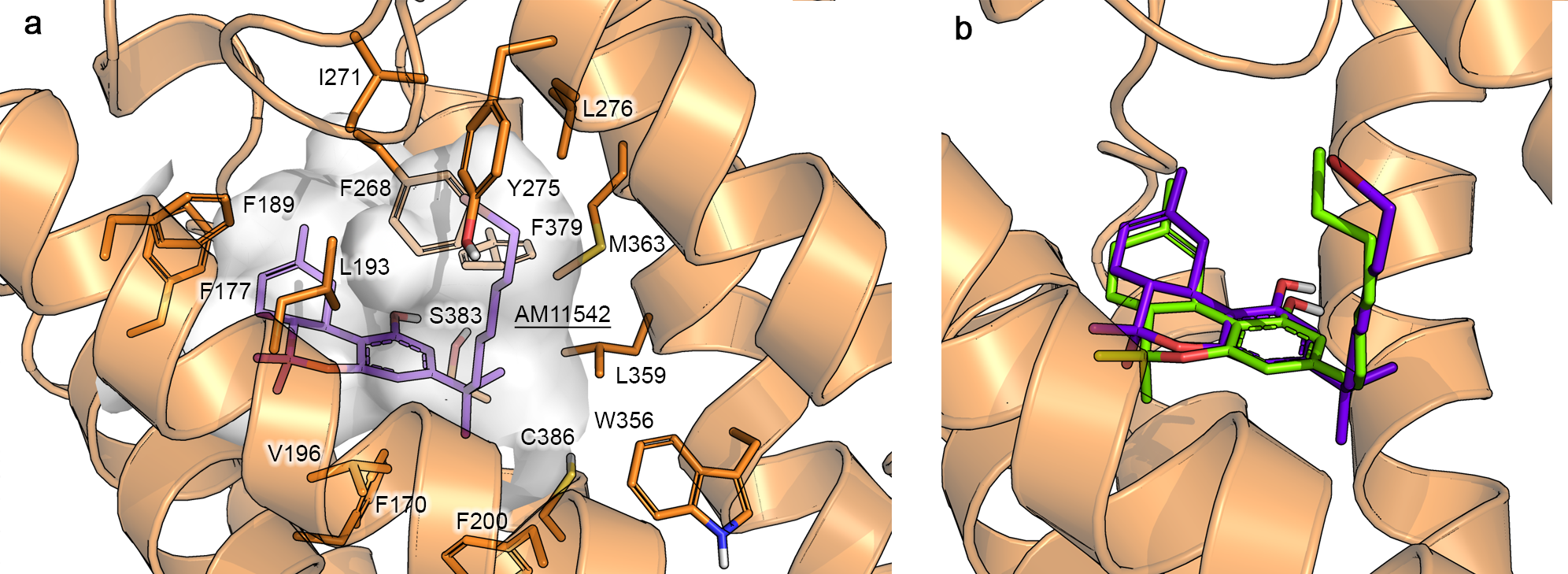 |
| --- |
| **Figure SI-6. a**, Crystal structure of hCB1 in complex with AM11542 agonist (PDB ID: 5XRA). **b**, Superposition of CB1-AM11542 (purple sticks) complex with the predicted binding mode of (-)-*trans*-Δ^9^-THCP (green sticks). The protein is represented in orange cartoon. Key amino acids are reported in orange sticks. Atoms are color coded: oxygen in red, bromine in ruby red. |

| 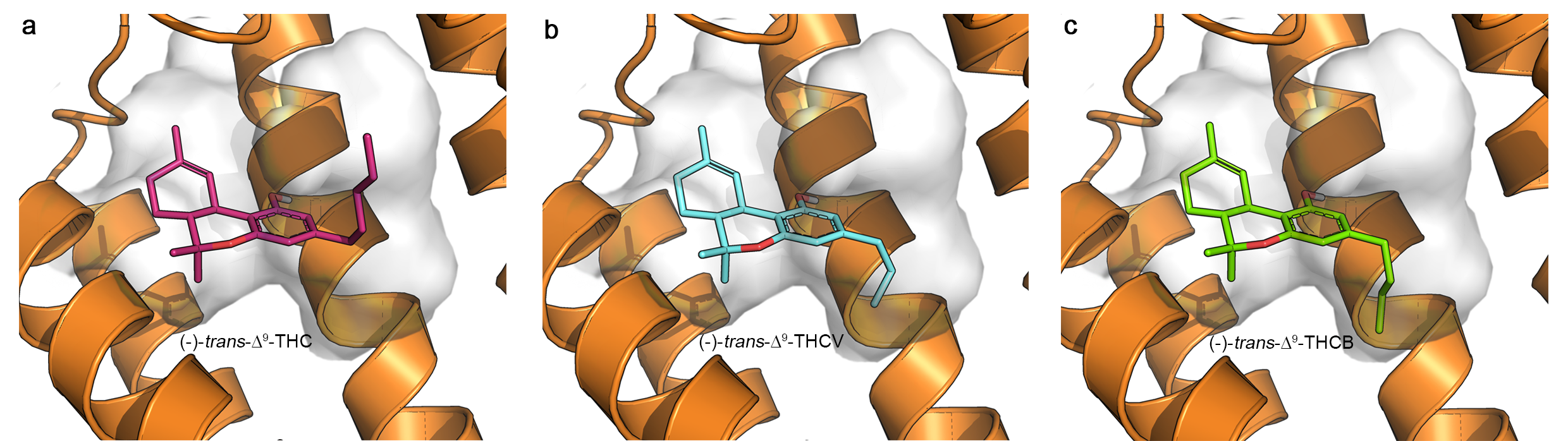 |
| --- |
| **Figure SI-7.** Predicted binding mode of (-)-*trans*-Δ^9^-THC (**a**, magenta sticks), (-)-*trans*-Δ^9^-THCV (**b,** cerulean sticks) and (-)-*trans*-Δ^9^-THCB (**c,** green sticks). |
